# CD39 dynamics in tuberculosis: a potential biomarker of immune dysregulation and T cell exhaustion

**DOI:** 10.3389/fimmu.2025.1601637

**Published:** 2025-08-11

**Authors:** Ling Hao, Qari Muhammad Imran, Nadeem Ullah

**Affiliations:** ^1^ Department of Pulmonary and Critical Care Medicine, Guangzhou First People’s Hospital, The Second Affiliated Hospital, School of Medicine, South China University of Technology, Guangzhou, China; ^2^ Institute of Clinical Medicine, Guangzhou First People’s Hospital, The Second Affiliated Hospital, School of Medicine, South China University of Technology, Guangzhou, China; ^3^ Department of Medical Biochemistry and Biophysics, Umeå University, Umeå, Sweden; ^4^ Department of Clinical Microbiology, Umeå University, Umeå, Sweden

**Keywords:** ENTPD1, CD39, tuberculosis, immune dysregulation, T cell exhaustion, inhibitory receptors

## Abstract

**Background:**

Tuberculosis (TB), caused by *Mycobacterium tuberculosis* (Mtb), remains a global health crisis complicated by immune dysregulation and T cell exhaustion. CD39, an ectonucleotidase generating immunosuppressive adenosine, is implicated in cancer and chronic infections, yet its spatiotemporal role in TB pathogenesis remains unclear.

**Methods:**

Multiple publicly available datasets were utilized to evaluate CD39 across TB disease stages, diverse infectious diseases and anti-TB treatment. Diagnostic accuracy was evaluated via ROC curves and combined signature analysis. Immune cell infiltration were analyzed using CIBERSORTx. Cytokine profiles and age-stratified associations were examined. Pathway enrichment analysis was performed by GSEA. Single-cell analysis of non-human primate granulomas assessed CD39’s temporal dynamics, utilizing Monocle 3 for CD39^+^ T-cell trajectory analysis.

**Results:**

CD39 was upregulated in active TB patients versus TB infection (TBI) and healthy controls (HC), correlating with older age, disease severity, and distinct expression patterns compared to other respiratory and systemic infections. CD39 demonstrated superior diagnostic accuracy over IFN-γ in distinguishing TB from TBI/HC and other respiratory diseases. Combining CD39 with TBX21 or GZMB further improved diagnostic specificity. High CD39 expression correlated with suppressed Th1 and elevated Th2/Th17/regulatory cytokines, alongside pronounced neutrophil infiltration. Age-stratified analysis revealed complex age-dependent associations of CD39 expression with various immune cell types. Single-cell analysis revealed declining CD39 transcriptional activity during prolonged infection despite expanded cellular distribution, linked to early T cell maturation followed by broader immunomodulatory shifts. Decreased CD39 expression with anti-TB treatment correlated with improved immune cell balance and resolved T cell exhaustion.

**Conclusion:**

CD39 is a critical regulator of immune exhaustion and neutrophil-driven inflammation in TB, with diagnostic and therapeutic potential. Targeting CD39 may provide a novel therapeutic strategy for TB.

## Introduction

Tuberculosis (TB), caused by *Mycobacterium tuberculosis* (Mtb), remains one of the deadliest infectious diseases worldwide, with an estimated 10.8 million new cases and 1.25 million deaths in 2023 (1). Despite advances in antimicrobial therapies (2), the emergence of multidrug-resistant (MDR) and extensively drug-resistant (XDR) strains, the limited efficacy of the only licensed vaccine, Bacillus Calmette-Guérin (BCG), combined with the challenges in controlling TB infection (TBI) necessitate a deeper understanding of the intricate host-pathogen interplay (3–5).

The success of Mtb as a pathogen depends on its ability to evade host immunity by establishing latent infections and manipulating immune cell functions (6). T lymphocytes, particularly CD4^+^ and CD8^+^ T cells, are critical for controlling intracellular bacterial growth via cytokine secretion (e.g., IFN-γ) and direct cytotoxic activity (7). However, persistent Mtb infection and the resulting chronic inflammatory environment often lead to T cell exhaustion, a state of T cell dysfunction that weakens protective immunity and accelerates disease progression (8). T cell exhaustion in TB is a complex and progressive phenomenon (9). It is characterized by functional impairment of CD4^+^ and CD8^+^ T cells, reduced production of key cytokines (e.g., IFN-γ, TNF-α, IL-2), and upregulation of multiple inhibitory receptors (IRs) (9). Multiple IRs have been elucidated their inhibitory mechanisms in experimental models of malignancies and chronic viral infections (10–13). For example, programmed cell death protein 1 (PD-1) recruited SHP-1/SHP-2 phosphatases via its immunoreceptor tyrosine-based inhibitory motif (ITIM), suppressing T-cell receptor (TCR) signaling through dephosphorylation of ZAP70 and PI3K/Akt pathways (10). Cytotoxic T-lymphocyte-associated protein 4 (CTLA-4) captured its ligands CD80 and CD86 from antigen-presenting cells (APCs) by a process of trans-endocytosis, leading to impaired CD28-dependent co-stimulation and thereby suppressing CD28-mediated T cell activation (11). T cell immunoreceptor with immunoglobulin and immunoreceptor tyrosine-based inhibitory domains (TIGIT) inhibited CD226-mediated immune activation by competitively binding to CD155 with higher affinity, and recruited SHIP1 phosphatase through its intracellular ITIM/ITT motifs, thereby suppressing PI3K/AKT and MAPK pathways in NK and T cells (12). Lymphocyte activation gene 3 (LAG-3) bound to the TCR-CD3 complex via its cytoplasmic EP motif, disrupting CD4/CD8 co-receptor interactions with the tyrosine kinase Lck, thereby suppressing proximal TCR signaling (13). Collectively, these IRs establish an immunosuppressive feedback loop that sustains T cell exhaustion, thereby contributing to the establishment of chronic infections like TB and accelerating disease progression.

The remarkable clinical success of immune checkpoint inhibitors in oncology, such as anti-PD-1 (pembrolizumab) and anti-CTLA-4 (ipilimumab) antibodies, which reinvigorated exhausted T cells in metastatic melanoma (14), lung cancer (15), and renal-cell carcinoma (16), provides a compelling rationale for adapting these strategies to TB. Preclinical evidence suggested translational potential of targeting some emerging IRs in TB. For example, T cell immunoglobulin and mucin domain-containing-3 (TIM-3) blockade enhanced IFN-γ, TNF and IL-2 production by both CD4^+^ and CD8^+^ T cells, and reduced pulmonary bacterial burdens in murine models of chronic Mtb infection (17). PD-1/PD-Ls blockade enhanced IFN-γ production by T cells in TB patients (18). However, the translational landscape is not uniformly optimistic. Other studies observed that PD-1 blockade exacerbated Mtb infection, increasing bacterial loads and mortality in murine and rhesus macaque models (19, 20). This paradoxical dichotomy might be due to the dual role of PD-1/PD-Ls to modulate a delicate equilibrium between immune activation and suppression (10). Thus, the timing and context of PD-1 blockade critically determine therapeutic outcomes. Importantly, similar complexity is observed with other IRs like TIGIT (21). Therefore, delineating the spatiotemporal dynamics of IRs across distinct disease phases is crucial for elucidating their mechanistic contributions to mycobacterial pathogenesis.

CD39, originally known as ectonucleoside triphosphate diphosphohydrolase-1 (ENTPD1), is a membrane-bound ectoenzyme that catalyzes the hydrolysis of extracellular ATP and ADP to AMP, which is subsequently converted to immunosuppressive adenosine by CD73 (22). This enzymatic cascade profoundly alters the extracellular nucleotide milieu, shifting it from a pro-inflammatory ATP-dominated state to an adenosine-rich immunosuppressive environment (22, 23). Preclinical studies highlight CD39 as a promising therapeutic target in cancer (24–26). For example, the CD39 inhibitor POM-1 (sodium polyoxotungstate) rescued exhausted T cells and increased CTL and NK cell-mediated cytotoxicity *in vitro* (24). Adenosine receptor antagonists like CPI-444 and SCH58261 could reverse T cell dysfunction and showed a synergistic effect with anti-PD-L1 and anti-CTLA-4 in mouse tumor models (25, 26). However, the role of CD39 in Mtb infection remains incompletely defined. Limited studies found that CD39 expression was increased on regulatory T cells (Tregs) and involved in mediating suppression of Th1 cell function in TB (27–29). These findings are primarily derived from small-scale observational studies, lacking comprehensive investigations from diverse ethnicities and multi-center samples. A comprehensive understanding of the spatiotemporal regulation of CD39 in TB pathogenesis is needed for its application in developing novel TB immunotherapy strategies.

In this study, we conducted integrated computational analyses of multi-center bulk and single-cell transcriptomic datasets to systematically investigate CD39 expression patterns across TB disease stages, diverse infections, and anti-TB treatment, evaluate its diagnostic utility in TB patients, and delineate its association with age-dependent immune alterations, impaired anti-TB immune responses and treatment recovery. Our findings indicated CD39 as a critical regulator of T cell exhaustion and neutrophil infiltration in TB and provided a scientific foundation for advancing immunotherapy strategies targeting IRs to restore functional anti-TB immunity.

## Materials and methods

### Data acquisition and processing

This study utilized publicly available datasets from the Gene Expression Omnibus (https://www.ncbi.nlm.nih.gov/geo/) database and the Single Cell Portal (https://singlecell.broadinstitute.org/). Specifically, eight bulk RNA-sequencing datasets related to TB were analyzed (GSE54992 (30), GSE28623 (31, 32), GSE83456 (33), GSE148036 (34), GSE184537 (35), GSE144127 (36), GSE31348 (37), and GSE19435 (38)). Datasets GSE54992, GSE31348 and GSE19435 provided longitudinal data from TB patients pre- and post-anti-TB treatment, enabling analysis of CD39 expression changes during treatment. For analysis of other infectious diseases, data were obtained from GSE6269 (39), GSE83148 (40), GSE159413, GSE217948 (41), GSE177477 (42), GSE161731 (43), GSE122737 (44), and GSE162760 (45). Details regarding the platform, sample size, organism and tissue type of each bulk RNA-seq dataset were summarized in **Table 1**. To track CD39 dynamics across different stages of Mtb infection, single-cell RNA sequencing (scRNA-seq) data were obtained from the SCP257 and SCP1749 studies via the Single Cell Portal (https://singlecell.broadinstitute.org/) and the GEO database (accession GSE200151) (46). These datasets profiled granulomas from non-human primates at both four weeks and ten weeks post-Mtb infection, allowing for the assessment of temporal changes in CD39 expression.

**Table 1:** Basic features of the datasets utilized in this study.

Accession	Platform	Sample	Organism	Tissue
GSE54992	GPL570	Active TB Pre-Treatment (n = 9) Active TB Post 3 Months of Treatment (n = 9) Active TB Post 6 Months of Treatment (n =9) TBI (n = 6) Healthy Controls (HC, n = 6)	Homo sapiens	PBMC
GSE28623	GPL4133	Active TB (n = 46) TBI (n = 25) HC (n = 37)	Homo sapiens	Whole blood
GSE83456	GPL10558	Pulmonary TB (PTB, n = 45) Extrapulmonary TB (EPTB, n = 47) Sarcoidosis (n = 49) HC (n = 61)	Homo sapiens	Whole blood
GSE148036	GPL21290	Lung TB Tissue (n = 5) Normal Lung (n = 5)	Homo sapiens	Lung tissue
GSE184537	GPL20301	DS-TB (n = 14) MDR-TB (n = 16) XDR-TB (n = 5)	Homo sapiens	Granuloma
GSE144127	GPL10558	PTB (n = 101) URTI (n = 8) LRTI (n = 11) Pneumonia (n = 61)	Homo sapiens	Whole blood
GSE31348	GPL570	Active TB Pre-Treatment (n = 27) Active TB post 4 Weeks of Treatment (n = 27) Active TB Post 26 Weeks of Treatment (n = 27)	Homo sapiens	Whole blood
GSE19435	GPL6947	Active TB Pre-Treatment (n = 7) Active TB post 2 Months of Treatment (n = 7) Active TB Post 12 Months of Treatment (n = 7) HC (n = 12)	Homo sapiens	Whole blood
GSE6269	GPL96	*E.coli* (n = 29) MSSA (n = 12) MRSA (n = 19) *S.pneumoniae* (n = 13) *Influenza A virus* (n = 18) HC (n=6)	Homo sapiens	PBMC
GSE83148	GPL570	HBV (n = 122) HC (n = 6)	Homo sapiens	Liver tissue
GSE159413	GPL18649	Untreated HBV (n = 12) NUC treated HBV (n = 13) HC (n = 19)	Homo sapiens	PBMC
GSE217948	GPL24676	COVID-19 (n = 175) HC (n = 62)	Homo sapiens	Whole blood
GSE177477	GPL23159	Symptomatic COVID-19 (n = 11) Asymptomatic COVID-19 (n = 18) HC (n = 18)	Homo sapiens	Whole blood
GSE161731	GPL24676	COVID-19 (n = 77) HC (n = 19)	Homo sapiens	Whole blood
GSE122737	GPL10558	Hookworm infection (n = 11) HC (n = 9)	Homo sapiens	Whole blood
GSE162760	GPL18573	Cutaneous leishmaniasis (n = 50) HC (n = 14)	Homo sapiens	Whole blood
GSE200151	GPL28212 GPL27448	4 weeks post-infection (n = 6) 10 weeks post-infection (n = 32)	Macaca fascicularis	Granuloma

### Diagnostic performance evaluation

The diagnostic potential of CD39 for TB was evaluated using Receiver Operating Characteristic (ROC) curve analysis. ROC curves were generated, and the Area Under the Curve (AUC) was calculated to assess the ability of CD39 to discriminate between different groups compared to a traditional biomarker IFN-γ. To evaluate combined diagnostic signatures of CD39 and TB-related markers (TBX21 and GZMB), binary logistic regression analysis was performed using SPSS software (version 26.0, IBM Corp., Armonk, NY, USA) to generate composite predictor probabilities. ROC analysis was subsequently applied to these logistic regression-derived combined probabilities to calculate AUC values and compare diagnostic performance against single markers. AUC values with 95% confidence intervals (CI) were computed for all individual and combined markers across specified comparison groups. The performance improvement of combined signatures versus CD39 alone was evaluated using DeLong’s test. A delta AUC (ΔAUC) of < 0.05 was considered not to substantially improve the diagnostic accuracy.

### Analysis of cytokine profiles

Cytokine expression across Th1 (IFN-γ, TNF-α, IL-2), Th2 (IL-4, IL-5, IL-13), Th17 (IL-17A, IL-21, IL-22), and regulatory (IL-6, IL-10, TGF-β) subsets was analyzed in TB patients using the GSE83456 dataset. Based on median ENTPD1 (CD39) expression, patients were stratified into CD39-high (top 50%) and CD39-low (bottom 50%) groups. Cytokine levels were compared between these groups. Pathway enrichment analysis was performed using Gene Set Enrichment Analysis (GSEA v4.4.0) as previously described (47), aiming to identify key pathways significantly enriched in the CD39-high expression group. Correlation analyses were conducted to assess the relationship between CD39 expression and the expressions of specific cytokines and neutrophil effector genes (ELANE and MPO).

### Analysis of immune cell infiltration and treatment-related changes

To characterize immune cell infiltration across TB disease states, bulk RNA-seq data from the GSE83456 (age-stratified analysis) and GSE28623 (TB/TBI/HC comparison) datasets were analyzed using CIBERSORTx (https://cibersortx.stanford.edu/), a deconvolution algorithm that quantifies immune cell subsets based on the LM22 leukocyte signature matrix – a validated reference containing gene expression profiles of 22 human immune cell types. The LM22 reference matrix includes signatures for granulocytes (eosinophils, neutrophils, mast cells), B cell subsets (naïve, memory, plasma cells), NK cells (resting/activated), phagocytes (monocytes, M0 macrophages), APCs (M1/M2 macrophages, resting/activated dendritic cells (DCs)), and T cell subsets (naïve/memory CD4^+^, CD8^+^, gamma delta T cells, Tregs, T follicular helper cells). CIBERSORTx was run in absolute mode with 1,000 permutations to estimate the relative proportions of immune cell subsets. Batch correction and normalization were applied to minimize technical variability across samples. Compositional immune cell profiles were visualized using stacked bar charts. Within the TB cohort, immune cell proportions were further compared between CD39-high (top 50%) and CD39-low (bottom 50%) groups, defined by median ENTPD1 (CD39) expression levels. Age-stratified correlation analyses assessed the relationship between CD39 expression and immune cell infiltration in Young (< 30 years), Mid (30–40 years), Late (40–50 years), and Older (≥ 50 years) groups. Longitudinal data from the GSE54992, GSE31348 and GSE19435 datasets were analyzed to investigate CD39 expression changes during TB treatment. Pathway enrichment analysis by GSEA identified key pathways enriched at different post-treatment time points. The GSE31348 dataset was also used to analyze the immune cell dynamics of successfully treated TB cases via CIBERSORTx. Finally, correlation analyses assessed the relationships between CD39 expression and proportions of various immune cell subsets throughout treatment.

### Single-cell transcriptomic analysis

To examine the temporal dynamics of CD39 expression during Mtb infection, scRNA-seq data from non-human primate granulomas were retrieved from the Single Cell Portal under study accessions SCP257 and SCP1749. These datasets profile immune cells in granulomas at two critical time points: 4 weeks (early infection) and 10 weeks (chronic infection) post-Mtb challenge. Initial analyses of the Single Cell Portal data focused on comparing CD39 (ENTPD1) expression between the 4-week and 10-week time points using existing cluster annotations. Uniform manifold approximation and projection (UMAP) was performed to visualize CD39 expression across immune cell clusters at both time points. Mean CD39 expression levels were calculated for each immune cell subset, using the Single Cell Portal’s analytical tools. For trajectory analysis, raw data from GSE200151 were downloaded and processed. Cells were filtered based on detected features (200–5000 genes) and mitochondrial content (< 5%, calculated using manually annotated *Macaca fascicularis* mitochondrial genes). Post-QC, 4-week and 10-week datasets were integrated. For data analysis, we applied Monocle 3 (48, 49). Batch correction was applied using the align_cds() function in Monocle 3 after PCA (50 components). Dimensionality reduction and clustering were performed on the aligned data using UMAP followed by Leiden clustering at a resolution of 1e-3. Clusters were annotated using existing “cell_type:ontology_label” metadata. To investigate CD39^+^ T cell dynamics, ENTPD1-expressing T cells were subsetted. Pseudotime trajectories were constructed using learn_graph() and order_cells(). CD39 expression dynamics were visualized along trajectories using UMAP embeddings, pseudotime density curves, and violin plots. Cluster-specific marker genes were identified using top_markers() (pseudo-R^2^ > 0.1, detection fraction > 10%).

### Statistical analysis

Statistical analysis of significant differences was acquired using the GraphPad Prism 8.0 software (GraphPad Software, Inc., La Jolla, CA, USA). Unpaired comparisons were assessed by a two-tailed *t*-test. Matched-sample comparisons were performed by a paired *t*-test. Multigroup analyses were carried out by a one-way ANOVA test, and Tukey’s multiple comparison test was used for further pair-wise comparison. Correlation analyses employed Spearman’s rank-order method with locally weighted scatterplot smoothing (loess) regression for nonparametric trend visualization. Diagnostic performance comparisons used DeLong’s test for ROC curves. Statistical significance was defined as *p* < 0.05.

## Results

### CD39 was highly expressed in TB patients and closely related to disease status and clinical characteristics

**Figure 1:**
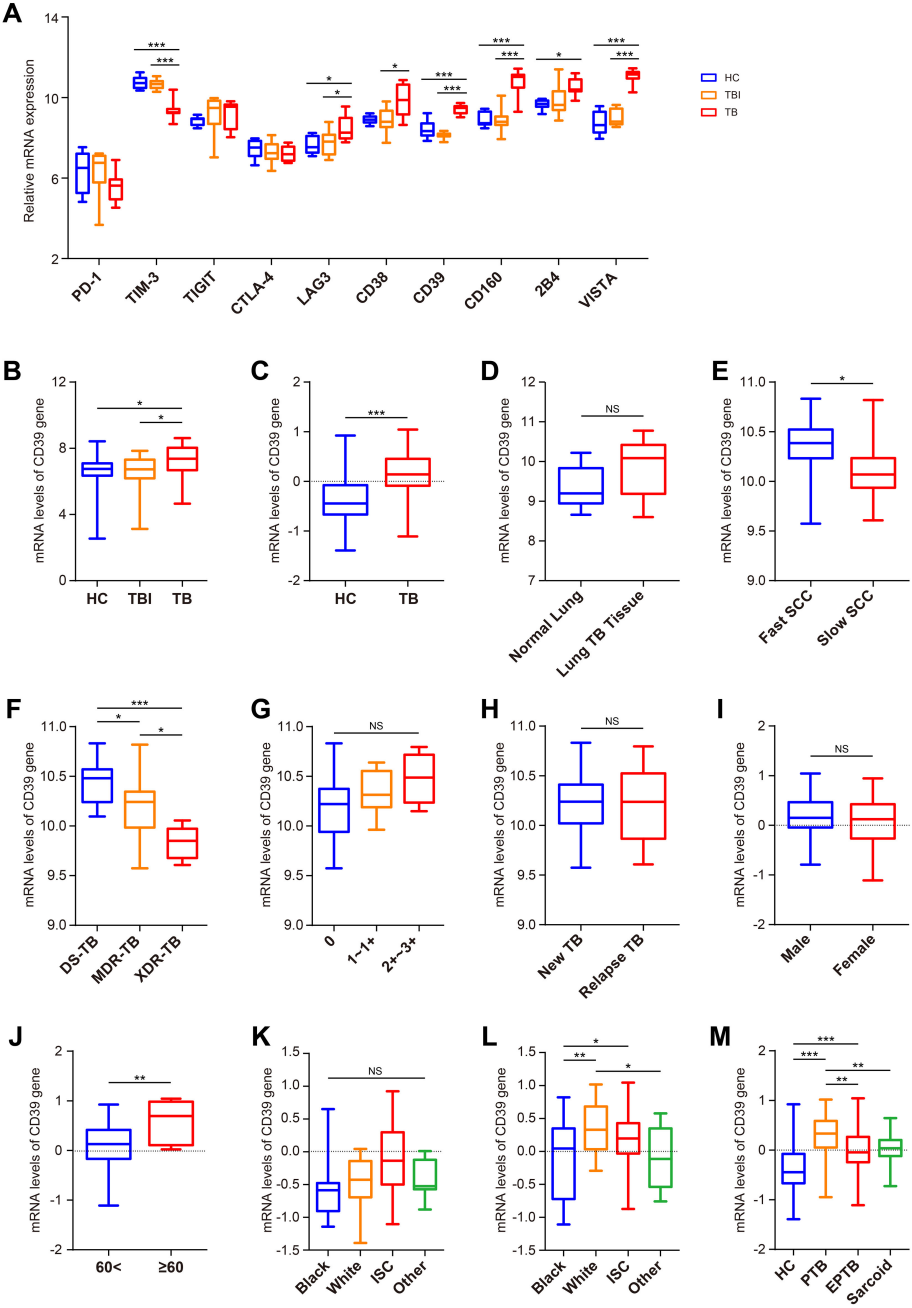
Expression analysis of CD39 across different Mtb infection states and demographic groups. **(A)** Expression patterns of CD39 alongside other T cell exhaustion-related genes in the HC, TBI, and active TB subgroups (GSE54992). **(B, C)** CD39 mRNA expression levels compared between: **(B)** HC, TBI, and active TB (GSE28623); **(C)** HC and active TB (GSE83456). **(D)** CD39 mRNA expression levels in normal versus TB lung tissues (GSE148036). **(E–H)** CD39 mRNA expression in TB patients (GSE184537) stratified by: **(E)** SCC (fast vs. slow); **(F)** drug sensitive (DS), MDR, and XDR-TB; **(G)** granuloma AFB grading; **(H)** new vs. relapse TB. **(I, J)** CD39 mRNA expression levels in active TB patients (GSE83456), compared by: **(I)** Gender; **(J)** Age (< 60 vs. ≥ 60 years). **(K, L)** CD39 mRNA expression levels compared across different ethnic groups of the HC **(K)** and TB **(L)** subgroups (GSE83456). **(M)** CD39 mRNA expression levels compared across different disease states (GSE83456). Statistical significance: *p < 0.05; **p < 0.01; ***p < 0.001; NS, not significant.

In this study, we evaluated the expression of ten T cell exhaustion markers in TB patients, TBI individuals and healthy controls (HC) using the PBMC-derived dataset GSE54992. Compared to the TBI and HC groups, LAG-3, CD39, CD160, and VISTA (V-domain Ig suppressor of T-cell activation) were upregulated in TB patients (all *p* < 0.05) (**Figure 1A**). Statistical validation across two additional whole-blood datasets (GSE28623 and GSE83456) confirmed elevated CD39 expression in TB patients (all *p* < 0.05) (**Figures 1B, C**). Notably, despite CD39 upregulation in blood compartments, analysis of lung tissue transcriptomes (GSE148036) revealed no significant difference in CD39 expression between TB lung tissues and adjacent normal tissues (**Figure 1D**). To assess the impact of CD39 levels on bacillary load and disease severity, we analyzed the GSE184537 dataset. Interestingly, fast converters (sputum culture conversion [SCC] < 2 months) exhibited higher CD39 levels in granulomas than slow converters (SCC > 2 months after treatment initiation) (*p* = 0.0228) (**Figure 1E**). Drug-resistant TB granulomas showed lower CD39 levels than drug-sensitive (DS) cases (all *p* < 0.05) (**Figure 1F**). However, no statistical differences were observed in CD39 levels among granulomas with different acid-fast bacilli (AFB) grading, or between granulomas of new TB patients and relapse TB patients (**Figures 1G, H**).

Next, leveraging the clinical data from GSE83456, we further assessed the impact of demographic and disease-specific factors on CD39 gene expression. No significant differences in CD39 levels were observed between male and female TB patients (**Figure 1I**). However, older patients (≥ 60 years) displayed relatively higher CD39 expression compared to younger patients (< 60 years) (*p* = 0.0052) (**Figure 1J**). While no race-associated differences in CD39 expression were detected in the HC group, White TB patients showed significantly higher CD39 levels than those of other ethnicities (**Figures 1K, L**). Furthermore, CD39 expression was notably increased in PTB compared to EPTB and sarcoidosis (all *p* < 0.01) (**Figure 1M**).

### CD39 exhibited distinct expression patterns across diverse infectious diseases

**Figure 2:**
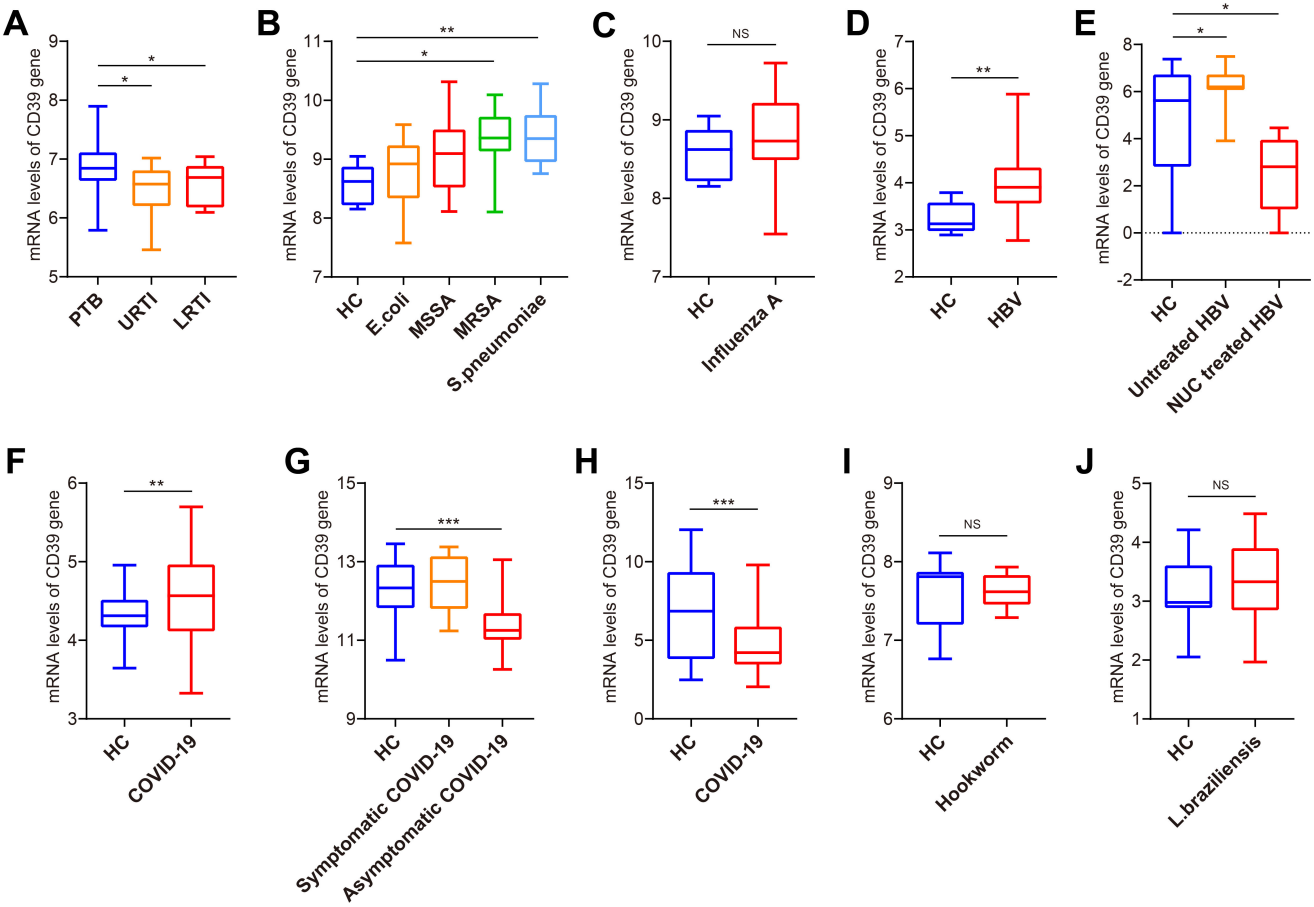
Expression analysis of CD39 in patients with TB and other infections. **(A)** CD39 mRNA expression in PTB, URTI and LRTI (GSE144127). **(B)** CD39 mRNA expression in HC and patients with bacterial infections (GSE6269). **(C)** CD39 mRNA expression in HC and patients with i*nfluenza A virus* infection (GSE6269). **(D)** CD39 mRNA expression levels between HC and HBV patients (GSE83148). **(E)** CD39 mRNA expression in HC, untreated and NUC treated HBV patients (GSE159413). **(F-H)** CD39 mRNA expression levels between HC and COVID-19 patients (GSE217948, GSE177477, GSE161731). **(I, J)** CD39 mRNA expression levels between HC and patients with parasitic infections: **(I)** Hookworm infection (GSE122737); **(J)** Cutaneous leishmaniasis (GSE162760). Statistical significance: *p < 0.05; **p < 0.01; ***p < 0.001; NS, not significant.

To evaluate disease specificity, we analyzed CD39 expression across various infectious disease cohorts. CD39 was significantly more elevated in PTB compared to upper and lower respiratory tract infections (URTI and LRTI) in GSE144127 (all *p* < 0.05) (**Figure 2A**). Analysis of GSE6269 revealed significantly elevated CD39 expression specifically in patients infected with methicillin-resistant *Staphylococcus aureus* (MRSA) or *Streptococcus pneumoniae* (all *p* < 0.05) (**Figure 2B**), while CD39 levels did not significantly differ from healthy controls (HC) in patients infected with *Escherichia coli*, methicillin-sensitive *S. aureus* (MSSA), or *Influenza A virus* (**Figures 2B, C**). Elevated CD39 expression was observed in both liver tissues and PBMCs from chronic hepatitis B virus (HBV) patients (all *p* < 0.05) (**Figures 2D, E**). However, CD39 levels in the coronavirus disease 2019 (COVID-19) patients were inconsistent compared to HC across three independent datasets (GSE217948, GSE177477 and GSE161731) (**Figures 2F–H**). Furthermore, CD39 levels in certain parasitic infections were similar to those observed in controls (**Figures 2I, J**). These findings suggested that CD39 upregulation is not a universal response to infection, but is selectively elevated in specific bacterial and viral infections like TB, MRSA, *S. pneumoniae*, and chronic hepatitis B.

### CD39 demonstrated superior accuracy in diagnosis TB

**Figure 3:**
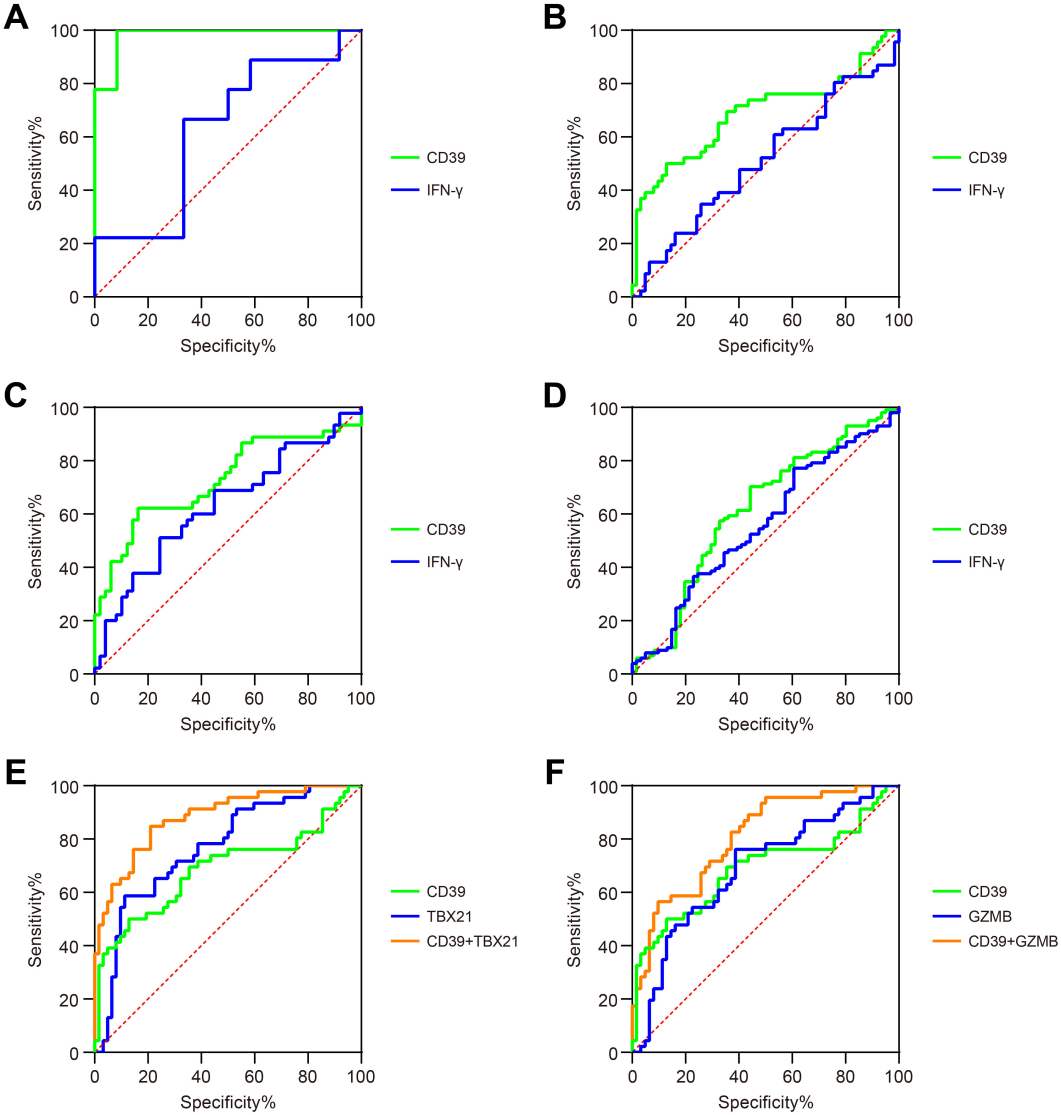
Diagnostic performance of CD39 in TB patients. ROC curves compared the ability of CD39 (green) and IFN-γ (blue) to discriminate between: **(A)** TB versus TBI and HC (GSE54492), **(B)** TB versus TBI and HC (GSE28623), **(C)** PTB versus sarcoidosis (GSE83456), and **(D)** PTB versus pneumonia (GSE144127). **(E)** ROC curves of CD39 (green), TBX21 (blue), and their combination signature (orange) for discriminating TB versus TBI and HC (GSE28623). **(F)** ROC curves of CD39 (green), GZMB (blue), and their combination signature (orange) for discriminating TB versus TBI and HC (GSE28623).

To evaluate the diagnostic potential of CD39 in TB, we analyzed publicly available GEO datasets, and found that CD39 demonstrated superior diagnostic performance compared to IFN-γ across multiple cohorts (**Figures 3A–D**). Specifically, in the GSE54992 dataset, CD39 achieved a notable AUC of 0.9815 (95% CI: 0.9347-1.000, *p* = 0.0002), effectively distinguishing TB patients from individuals with TBI and HC (**Figure 3A**). Similarly, in the GSE28623 dataset, CD39 yielded an AUC of 0.6886 (95% CI: 0.5808-0.7965, *p* = 0.0008) (**Figure 3B**). These values significantly exceeded those of IFN-γ (AUCs: 0.6269 and 0.5102, respectively). Clinical diagnostic validation using the GSE83456 dataset further highlighted CD39’s utility, yielding an AUC of 0.7270 (95% CI: 0.6214-0.8365, *p* = 0.0002) for distinguishing PTB from sarcoidosis, compared to IFN-γ’s lower accuracy (AUC = 0.6286) (**Figure 3C**). Additionally, CD39 exhibited enhanced diagnostic value than IFN-γ (AUC = 0.5582) in differentiating PTB from pneumonia, with an AUC of 0.6165 (95% CI: 0.5228-0.7072, *p* = 0.0143) (**Figure 3D**).

To enhance diagnostic specificity, we evaluated a combined signature of CD39 and TB-antigen-responsive genes TBX21 and GZMB. In the GSE28623 cohort, combined CD39 and TBX21 signature achieved an AUC of 0.8829 (95% CI: 0.8196-0.9462, *p* < 0.0001) for discriminating TB from TBI and HC, significantly outperforming CD39 alone (AUC = 0.6886) or TBX21 alone (AUC = 0.7686, *p* < 0.0001) (**Figure 3E**). A similar improvement was observed with the combined CD39 and GZMB signature (AUC = 0.8072, 95% CI: 0.7263-0.8880, *p* < 0.0001) for the same comparison, again significantly exceeding the performance of CD39 alone or GZMB alone (AUC = 0.6879, *p* = 0.0009) (**Figure 3F**). However, these combined signatures failed to substantially improve diagnostic accuracy in distinguishing PTB from sarcoidosis or pneumonia compared to CD39 alone (ΔAUC < 0.05, data not shown). These findings collectively highlighted CD39 as a promising biomarker for TB diagnosis, offering improved diagnostic accuracy, particularly when combined with other TB-related markers.

### CD39 expression was associated with cytokine imbalance in TB patients

**Figure 4:**
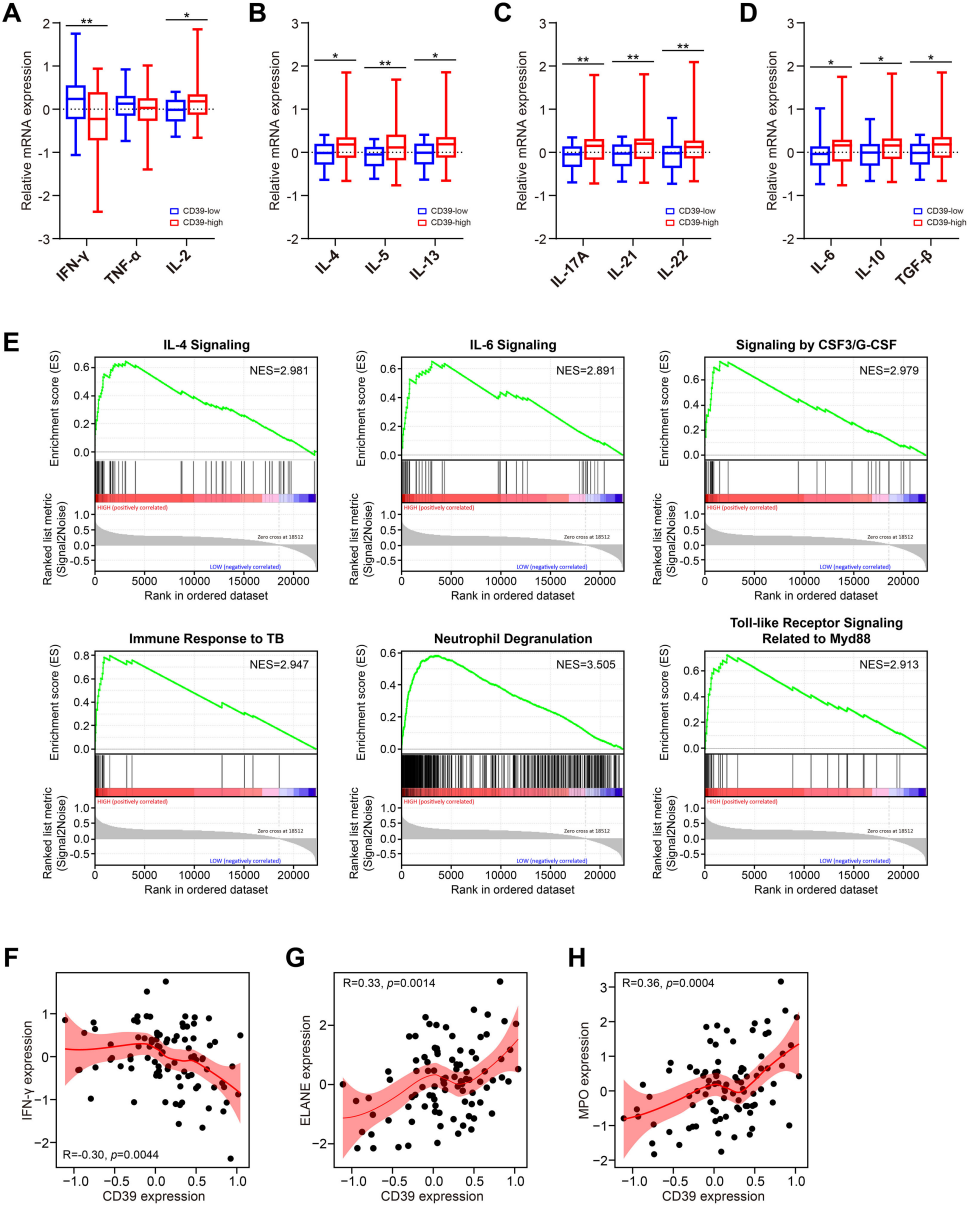
Association of CD39 expression with cytokine responses in TB patients. **(A–D)** Cytokine levels in TB patients stratified by CD39 expression (high vs. low): **(A)** Th1, **(B)** Th2, **(C)** Th17, and **(D)** regulatory cytokines. **(E)** Significantly enriched pathways identified by GSEA in CD39-high TB patients. **(F-H)** Correlation between CD39 expression and levels of IFN-γ **(F)**, ELANE **(G)**, and MPO **(H)** in TB patients.

Cytokine-mediated immune responses are fundamental to anti-TB immunity. Dysregulation of Th1, Th2, Th17, or regulatory cytokine networks can profoundly alter disease trajectories in Mtb infection (50). Hence, we explore the association between CD39 expression and these critical cytokine profiles in TB patients using the GSE83456 dataset. Patients were divided into high and low CD39 expression groups based on a median split (top 50% vs. remaining 50%). We observed that patients with high CD39 expression displayed a significantly altered cytokine balance compared to those with low CD39 expression, characterized by: lower levels of the Th1 cytokine IFN-γ (**Figure 4A**), and elevated levels of the Th2 cytokines IL-4, IL-5 and IL-13 (**Figure 4B**), the Th17 cytokines IL-17A, IL-21, and IL-22 (**Figure 4C**), and the regulatory cytokines IL-6, IL-10 and TGF-β (**Figure 4D**) (all *p <*0.05). IL-2 was also significantly elevated in the CD39-high group (*p* = 0.0169) (**Figure 4A**). Pathway enrichment analysis in the CD39-high group revealed significant enrichment of the IL-4, IL-6, and CSF3/G-CSF signaling pathways, along with pathways related to the immune response to TB, neutrophil degranulation, and toll-like receptor signaling related to Myd88 (**Figure 4E**) (**Supplementary Table S1**). Furthermore, correlation analyses indicated a negative relationship between CD39 expression and IFN-γ (**Figure 4F**). CD39 expression showed significant positive correlations with neutrophil effector genes ELANE (neutrophil elastase) and MPO (myeloperoxidase) (**Figures 4G, H**). These findings suggested that CD39 might be involved in shaping the cytokine milieu and neutrophil effector functions in TB patients, potentially influencing the balance between protective Th1 immunity and detrimental immunopathology driven by Th2, Th17, regulatory cytokines and neutrophil-mediated inflammation.

### CD39 exhibited age-dependent immunoregulatory roles in TB patients

**Figure 5:**
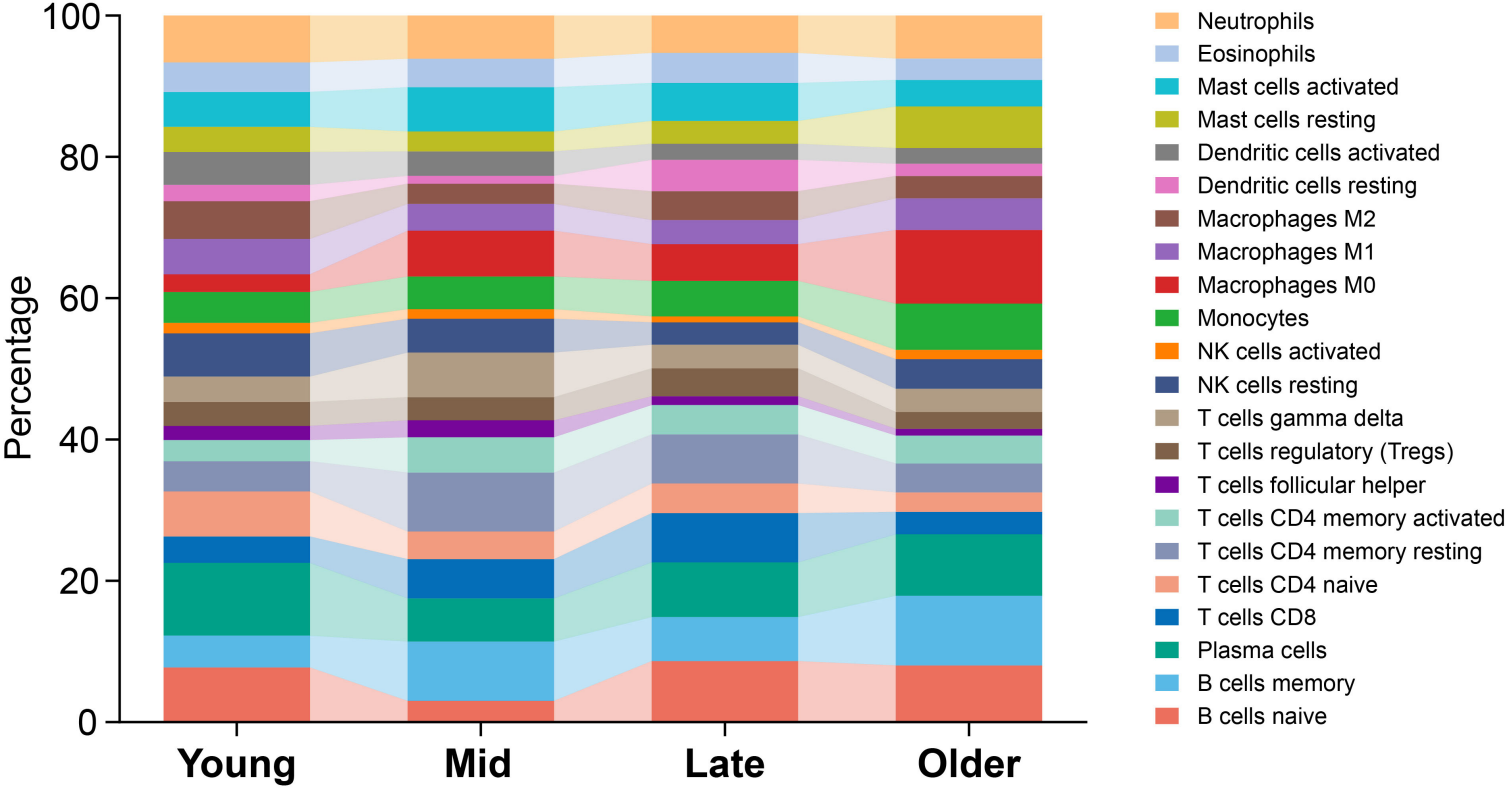
Age-related distribution of immune cell subsets in TB patients. Average proportions were presented for Young, Mid, Late, and Older TB patients, with age ranges defined as < 30, 30-40, 40-50, and ≥ 50 years, respectively.

**Figure 6:**
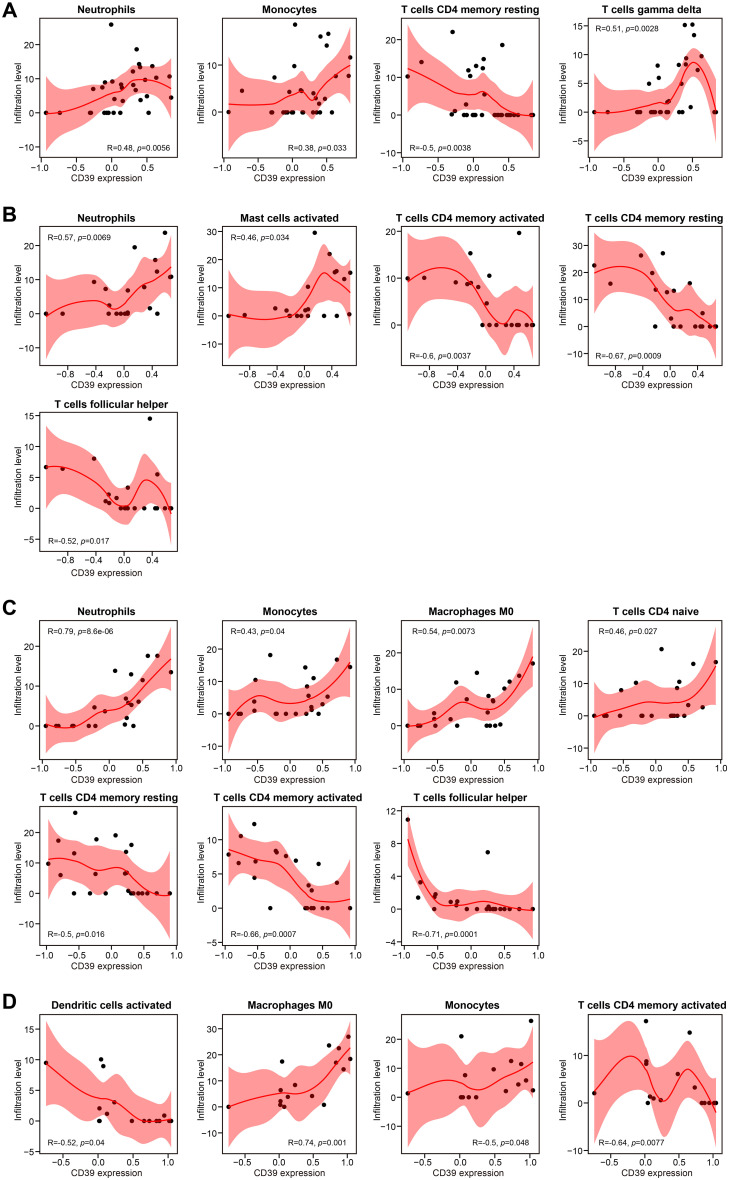
Correlation analysis of CD39 expression and immune cell abundance, stratified by age in TB patients. Results were presented for **(A)** Young, **(B)** Mid, **(C)** Late, and **(D)** Older age groups.

Given the age-dependent increase in CD39 expression (**Figure 1J**), we investigated age-related immunoregulatory roles of CD39 in TB patients using the GSE83456 dataset. Active TB patients were stratified into Young (< 30 years), Mid (30–40 years), Late (40–50 years), and Older (≥ 50 years) adult groups. Of the immune cell subsets examined (**Figure 5**), only M0 macrophages significantly increased with age (*p* = 0.0040), peaking in Older adults. All other cell types showed no significant age-related differences. Age-stratified correlation analysis revealed that CD39 exhibited both consistent and age-specific associations with immune cell infiltration (**Figure 6**). Specifically, CD39 positively correlated with neutrophils in Young, Mid, and Late adults (**Figures 6A–C**), but negatively correlated with memory CD4^+^ T cells: resting memory CD4^+^ T cells in the same age groups, and activated memory CD4^+^ T cells in Mid, Late, and Older adults (**Figures 6B–D**). Monocytes positively correlated with CD39 in Young, Late, and Older adults (**Figures 6A, C, D**), while follicular helper T cells negatively correlated in Mid and Late adults (**Figures 6B, C**). In Late and Older adults, CD39 positively correlated with M0 macrophages (**Figures 6C, D**). Age-specific correlations included positive associations with gamma delta T cells (Young), activated mast cells (Mid), and naïve CD4^+^ T cells (Late), and a negative association with activated DCs (Older) (**Figures 6A–D**). These findings suggested that CD39 had both consistent and age-dependent associations with the immune landscape in active TB, influencing neutrophils, memory T cells, macrophages, and monocytes in distinct age groups.

### CD39 expression was associated with neutrophil infiltration in TB patients

**Figure 7:**
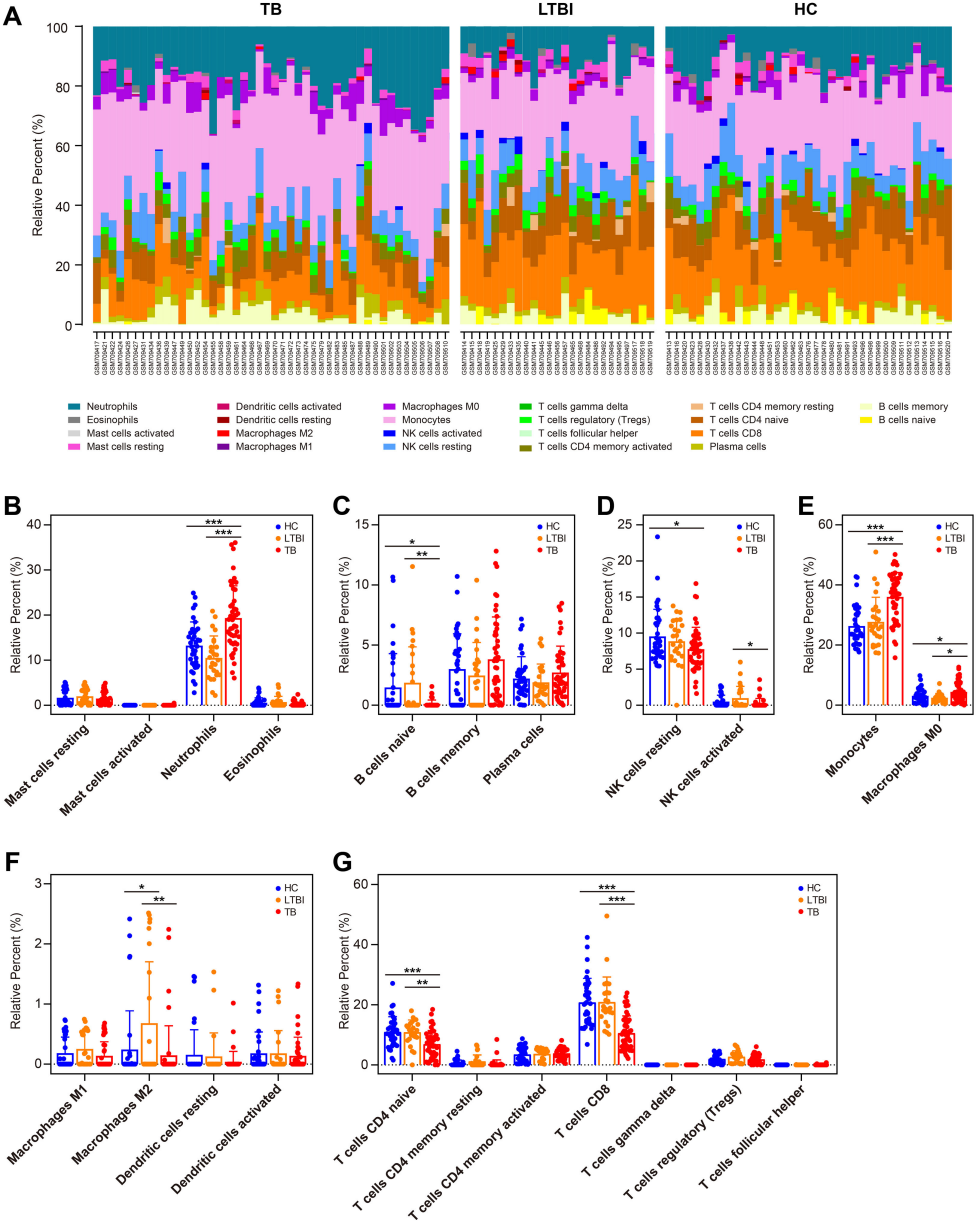
Immune cell profiling in HC, TBI, and active TB patients. **(A)** Compositional immune cell profiles (stacked bar charts) across HC, TBI, and TB groups via CIBERSORTx. **(B–H)** Comparison of infiltration levels of specific immune cell subsets: granulocytes **(B)**, B cell subsets **(C)**, NK cells **(D)**, phagocytes **(E)**, APCs **(F)**, and T cell subsets **(G)**. Statistical significance: *p < 0.05; **p < 0.01; ***p < 0.001.

To characterize the immune environment in TB, we compared immune cell subset infiltration among TB, TBI, and HC groups using the GSE28623 dataset. Our analysis revealed that immune cell expression patterns in TBI and HC were largely congruent, contrasting with TB patients, who showed measurable but incomplete divergence, potentially reflecting disease-associated immune dysregulation (**Figure 7A**). Compared to TBI and HC, TB patients displayed elevated numbers of neutrophils, monocytes, and M0 subtype macrophages (all *p* < 0.05, **Figures 7B, E**), while showing reduced counts of naive B cells, CD8^+^ T cells, and naive CD4^+^ T cells (all *p* < 0.05, **Figures 7C, G**). In addition, TB patients had lower number of activated NK cells and M2 subtype macrophages compared to TBI individuals (all *p* < 0.05, **Figures 7D, F**).

**Figure 8:**
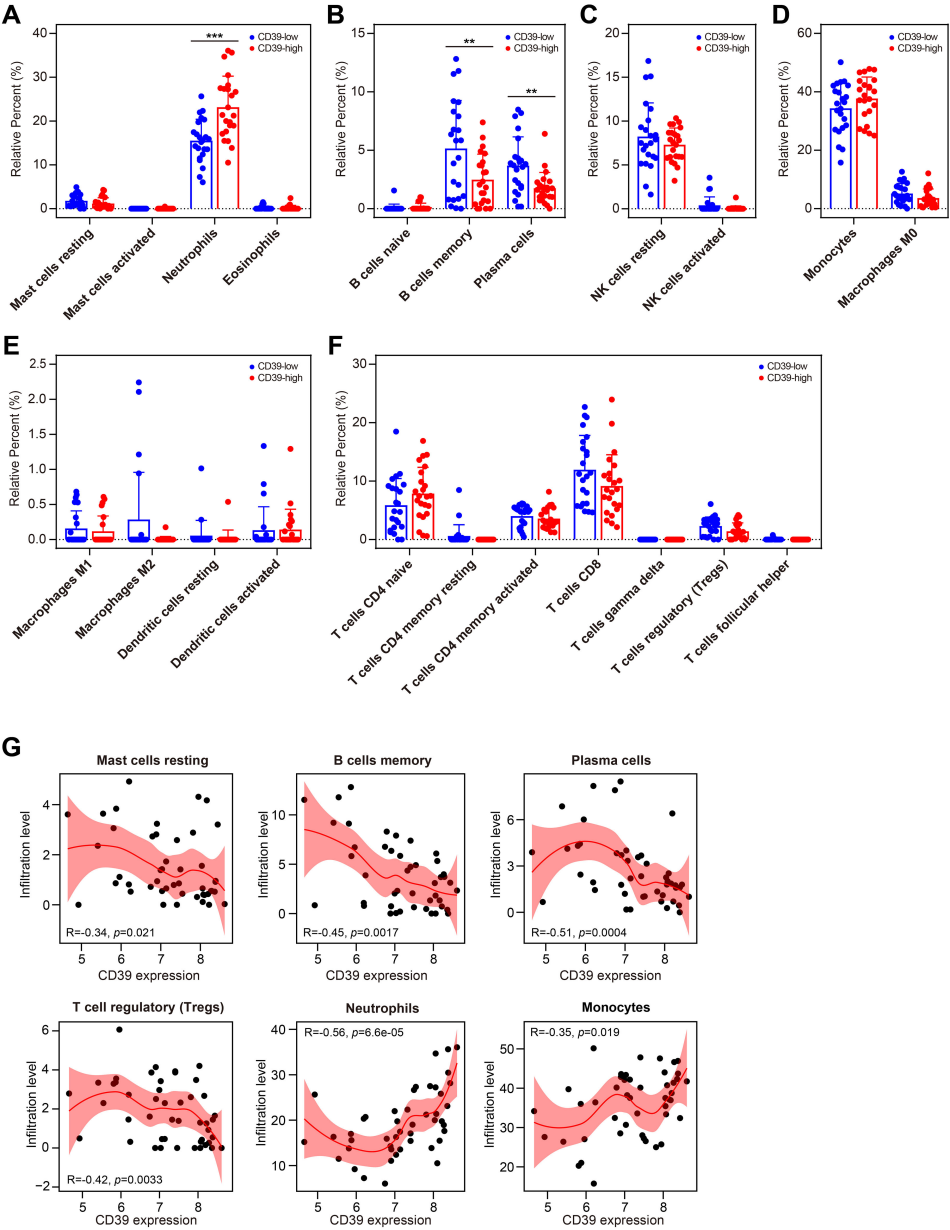
Relationship between CD39 expression and immune cell populations in TB. **(A-G)** Infiltration levels of specific immune cell subsets compared between CD39-high and CD39-low groups: granulocytes **(A)**, B cell subsets **(B)**, NK cells **(C)**, phagocytes **(D)**, APCs **(E)**, and T cell subsets **(F)**. **(G)** Correlation between CD39 expression and the abundance of the immune cells in TB patients. Statistical significance: **p < 0.01; ***p < 0.001.

We further investigated the impact of CD39 expression on the immune cell landscape in TB patients. The 46 TB samples in GSE28623 were divided into two groups based on CD39 expression, with 23 samples in the high-expression group and 23 samples in the low-expression group. Notably, TB patients with high CD39 expression exhibited pronounced neutrophil infiltration (**Figure 8A**). Conversely, in the TB group with low CD39 expression, the levels of memory B cells and plasma cells were increased (**Figure 8B**). No significant differences were found in the infiltration of innate lymphoid cells, phagocytes, APCs, or T cell subsets between the CD39-defined subgroups (**Figures 8C–F**). Correlation analyses revealed a negative correlation between CD39 expression and resting mast cells, memory B cells, plasma cells, and Tregs, while a positive correlation was observed between CD39 expression and neutrophils and monocytes (**Figure 8G**). These findings suggested that CD39 might influence immune cell dynamics in TB, potentially contributing to disease pathogenesis by disrupting the balance of innate and adaptive immune responses.

### Altered CD39 transcriptional activity drove broad immune cell alterations and T cell differentiation during prolonged Mtb infection

**Figure 9:**
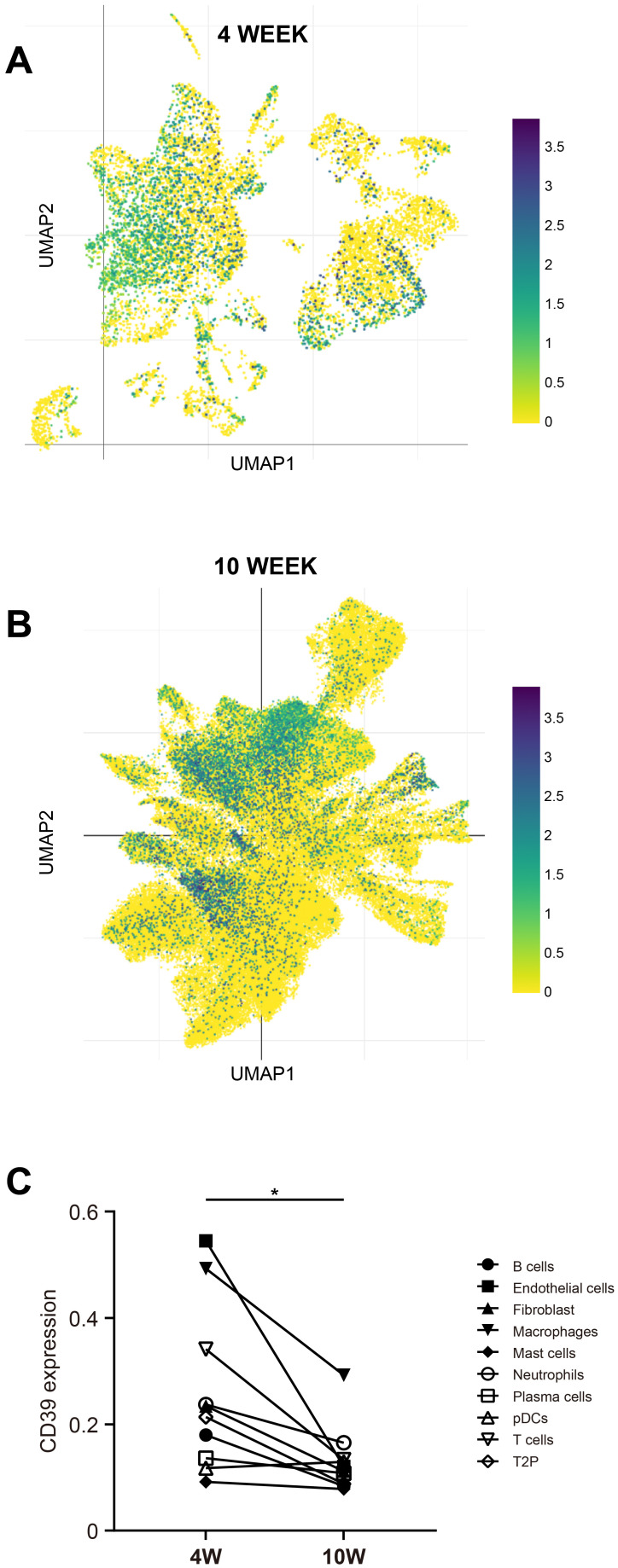
CD39 expression changed with the duration of Mtb infection. UMAP visualization of scRNA-seq data (SCP257 and SCP1749 studies) illustrated CD39 expression patterns at two time points: **(A)** 4 weeks post-infection and **(B)** 10 weeks post-infection (colored by CD39 expression). **(C)** Mean CD39 expression levels in different immune cell subsets at 4 and 10 weeks post-Mtb infection were compared. Statistical significance: *p < 0.05.

We further determine the impact of Mtb infection duration on CD39 expression across different immune cell types using publicly available scRNA-seq data from the SCP257 and SCP1749 studies. These datasets profile granulomas from non-human primates at two time points: four weeks and ten weeks post-infection. The specific positions of different immune cells within the UMAP space were shown in **Supplementary Figure S1**. Clustering analysis of immune cell subpopulations revealed that CD39 was primarily expressed in macrophages, T cells, and endothelial cells during the early (four-week) stage of infection (**Figure 9A**). By ten weeks post-infection, a progressive increase in the distribution density of CD39 was observed, encompassing a broader range of immune cell subpopulations: macrophages, T cells, B cells, endothelial cells, mast cells, neutrophils, and plasma cells (**Figure 9B**). Intriguingly, despite this expanded distribution, the mean mRNA levels of CD39 within individual immune cell types decreased as the infection progressed (*p* = 0.0113, **Figure 9C**).

**Figure 10:**
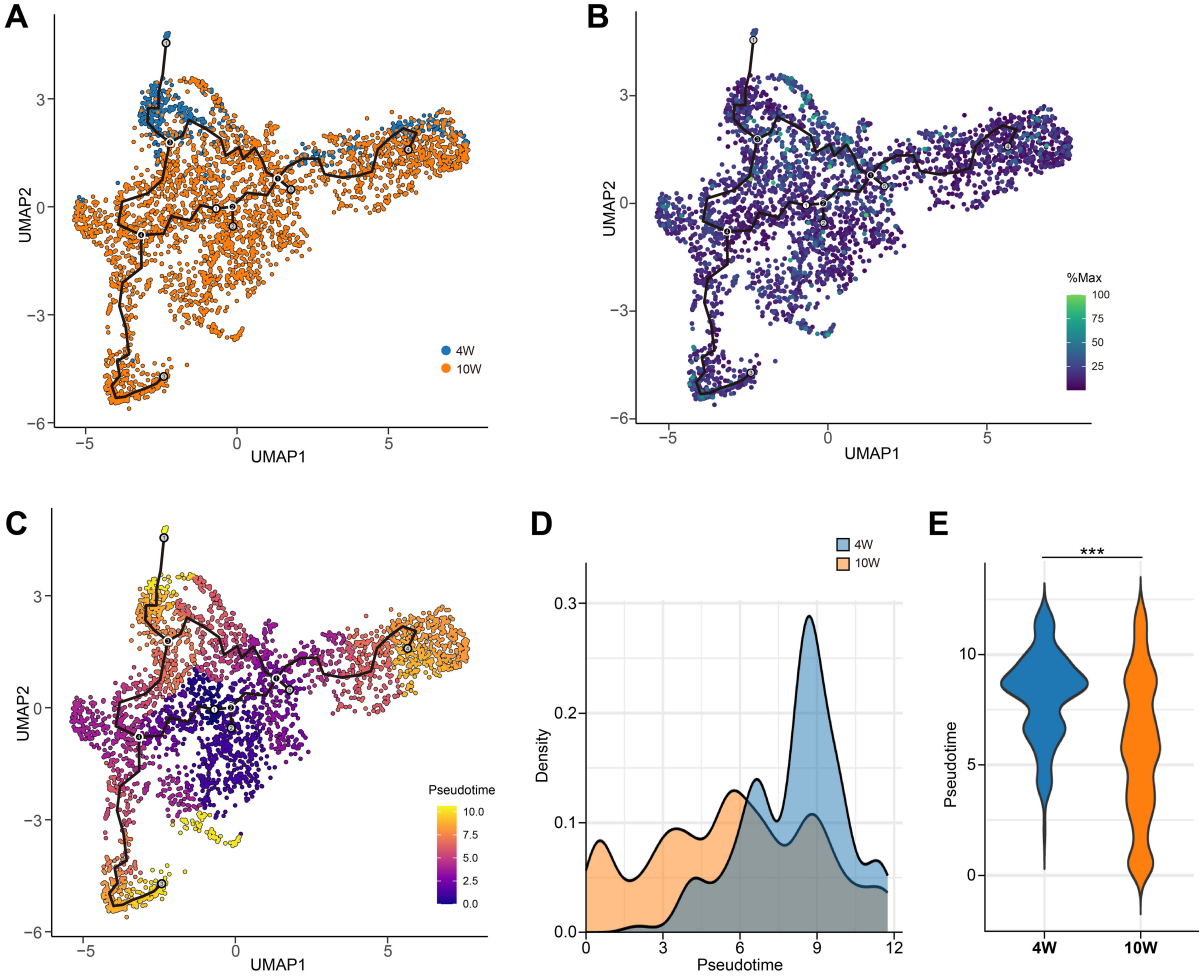
Pseudotemporal dynamics of CD39^+^ T cells during Mtb infection in granulomas. **(A)** UMAP visualization of CD39^+^ T cells from combined 4-week and 10-week post-Mtb infection granulomas (GSE200151). **(B)** CD39 (ENTPD1) expression levels overlaid on the UMAP. **(C)** Pseudotime values for CD39^+^ T cells mapped onto the UMAP. **(D, E)** Density plot **(D)** and violin plot **(E)** comparing the distribution of pseudotime values at 4 and 10 weeks post-Mtb infection. Statistical significance: ***p < 0.001.

To further characterize the role of CD39, particularly its influence on T cell dynamics, we performed trajectory analysis on combined 4- and 10-week data. Immune cell positions and their CD39 expression patterns, visualized within the UMAP, were detailed in **Supplementary Figure S2**. Trajectory analysis of CD39^+^ T cells revealed a developmental progression from early-state (purple clusters, center of UMAP) to mature T cells (orange-yellow clusters, periphery of UMAP) (**Figures 10A–C**). Critically, pseudotime analysis revealed distinct patterns at different time points: at 4 weeks post-infection, CD39^+^ T cells were predominantly found in a terminal differentiation state, whereas at 10 weeks, they exhibited a more uniform distribution of pseudotime values, suggesting a relative plateau in their development (**Figures 10D, E**). Given CD39’s high expression at the early stage (4-week) of Mtb infection, its elevated expression might be linked to accelerated T cell maturation, leading to rapid terminal differentiation. These findings suggested that while CD39 expression became more widespread across immune cell populations over time, its transcriptional activity within specific cells might decline, coinciding with its transition from a driver of T cell maturation in early infection to a more muted role at later stages.

### CD39 downregulation correlated with reversal of T cell exhaustion and enhanced immune competence in treated TB

**Figure 11:**
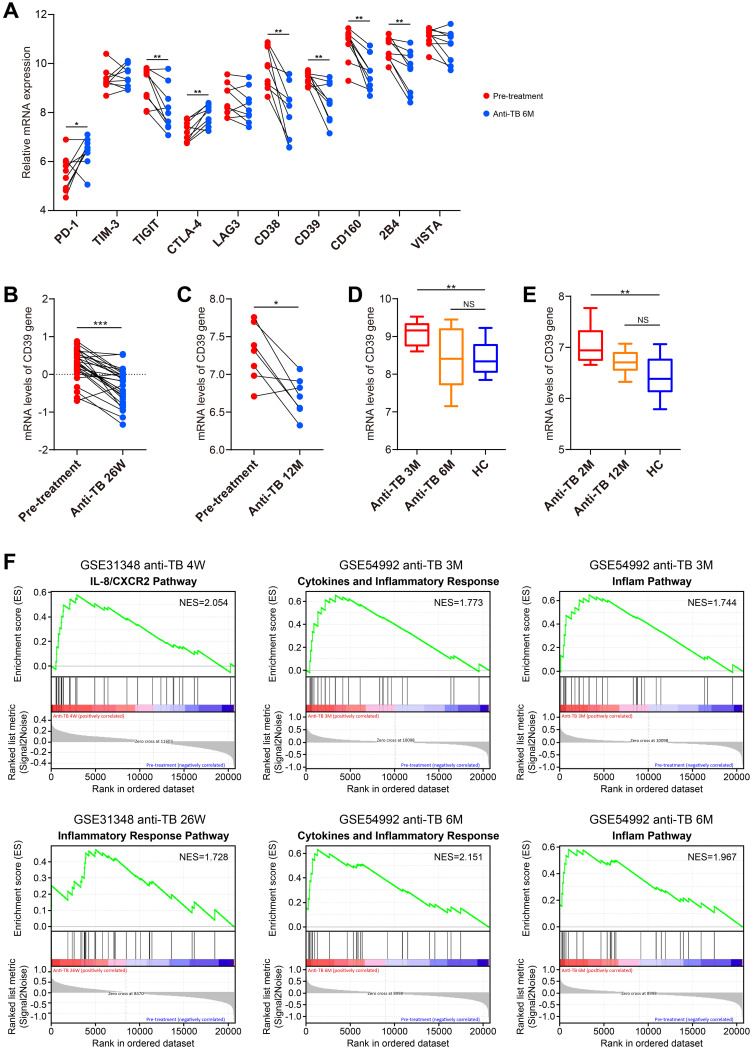
Dynamic changes in CD39 expression and pathway modulation during anti-TB treatment. **(A)** Temporal changes in CD39 expression compared to other T cell exhaustion markers between pre-treatment and 6-month post-treatment TB patient samples (GSE54992). **(B–E)** CD39 mRNA expression levels compared between: **(B)** pre-treatment and 26-week post-treatment (GSE31348); **(C)** pre-treatment and 12-month post-treatment (GSE19435); **(D)** 3-month, 6-month post-treatment and HC (GSE54992); **(E)** 2-month, 12-month post-treatment and HC (GSE19435). **(F)** Significantly enriched pathways identified by GSEA at each post-treatment stage. Statistical significance: *p < 0.05; **p < 0.01; ***p < 0.001; NS, not significant.

We conducted an in-depth investigation to analyze changes of CD39 expression before and after anti-TB drug treatment. Notably, 6 months post-treatment, the expression levels of CD39 and several other T cell exhaustion markers, including TIGIT, CD38, CD160, and 2B4, were significantly reduced (*p* < 0.05) compared to their pre-treatment levels in the GSE54992 dataset (**Figure 11A**). This reduction in CD39 expression was further confirmed in the two additional datasets: GSE31348 at 26 weeks and GSE19435 at 12 months post-treatment (all *p* < 0.05, **Figures 11B, C**). CD39 expression in TB patients completing ≥ 6 months of therapy returned to levels comparable to HC individuals (**Figures 11D, E**). In contrast, chronic HBV patients receiving long-term nucleoside analogue (NUC) therapy exhibited significantly lower CD39 than HC individuals (**Figure 2E**). Pathway enrichment analysis revealed early enrichment of inflammatory modulation pathways (4 weeks to 26 weeks post-treatment versus baseline) (**Figure 11F**) (**Supplementary Tables S2**–**5**), and later enrichment of nucleic acid metabolism pathways (e.g., RNA processing, DNA replication) after prolonged anti-TB therapy (12 months) (**Supplementary Table S6**).

**Figure 12:**
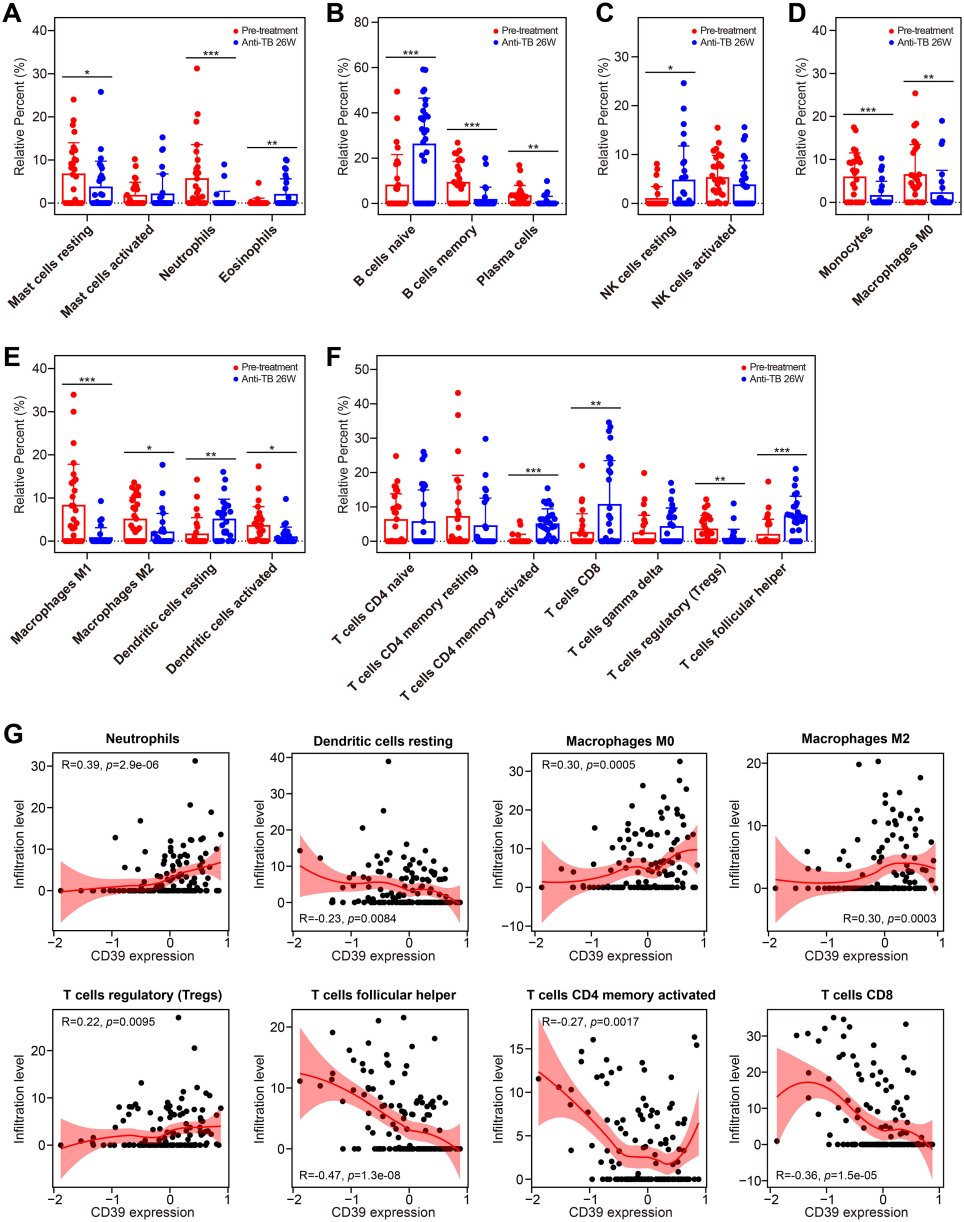
Impact of anti-TB treatment on immune cell infiltration and CD39 correlation. **(A-G)** Treatment-induced changes in the infiltration of granulocytes **(A)**, B cell subsets **(B)**, NK cells **(C)**, phagocytes **(D)**, APCs **(E)**, and T cell subsets **(F)** (pre-treatment vs. 26 weeks). **(G)** Correlation between CD39 expression and overall immune cell abundance during TB treatment. Statistical significance: *p < 0.05; **p < 0.01; ***p < 0.001.

Using the GSE31348 dataset, we further analyzed immune cell dynamics. Successfully treated cases displayed elevated numbers of eosinophils, naive B cells, resting NK cells and DCs, activated memory CD4^+^ T cells, CD8^+^ T cells, and T follicular helper cells (all *p* < 0.05, **Figures 12A–C, E, F**), while showing decreased counts of resting mast cells, neutrophils, memory B cells, plasma cells, monocytes, macrophages (M0/M1/M2 subtypes), and Treg cells (all *p* < 0.05, **Figures 12A, B, D–F**). Critically, CD39 expression correlated inversely with resting DCs, activated memory CD4^+^ T cells, CD8^+^ T cells, and T follicular helper cells, while showing positive correlations with neutrophils, macrophages (M0/M2 subtypes), and Tregs throughout treatment (**Figure 12G**). These findings suggested that CD39 reduction post-treatment reflected systemic immune restoration, with its correlations to effector T cell activation and immunosuppressive cell contraction highlighting the resolution of T cell exhaustion and the re-establishment of antimicrobial immunity.

## Discussion

Despite advancements in vaccine development, drug discovery, and molecular diagnostics, TB control remains hampered by the evolutionary arms race between Mtb and the human immune system. Over millennia, Mtb has evolved sophisticated mechanisms to hijack host inhibitory pathways, enabling it to subvert immune defenses and establish chronic infection (6). These include exploiting T cell exhaustion marked by the sustained expression of IRs and ligand-driven suppression of T cell activation, thereby limiting the host’s ability to clear the bacteria (9, 17, 18, 29). While T cell exhaustion and inhibitory receptor upregulation are hallmarks of chronic infections, their spatiotemporal regulation in TB, particularly within granulomas, remains poorly defined. In the current study, we systematically investigated the role of CD39, a critical ectonucleotidase in the ATP-adenosine pathway, across TB disease stages, diverse infectious diseases, and anti-TB treatment. We demonstrated that CD39 was significantly upregulated in active TB patients compared to TBI and HC individuals, correlating with disease status and clinical characteristics, and its expression patterns differed from other respiratory infections and various bacterial and viral infections. CD39 exhibited superior diagnostic accuracy over IFN-γ across multiple cohorts, with further improvement when combined with a TB-antigen-responsive gene (TBX21 or GZMB) for distinguishing TB from TBI/HC, highlighting its potential as a biomarker. Mechanistically, elevated CD39 expression was associated with suppressed Th1 cytokines, amplified Th2/Th17/regulatory cytokines, and pronounced neutrophil infiltration with correlated upregulation of neutrophil effector genes (ELANE and MPO). Age-stratified analysis revealed complex age-dependent immunoregulatory roles for CD39, impacting neutrophils, memory T cells, macrophages, and monocytes in distinct age groups. Single-cell RNA-seq data indicated dynamic CD39 transcriptional activity during prolonged Mtb infection: an early burst promoting T cell maturation, followed by a shift towards broader immune cell expression coupled with decreased activity, suggesting a transition from T cell differentiation to broader immunomodulatory roles. Longitudinal analysis further revealed CD39 downregulation post-treatment correlated with the reversal of T cell exhaustion and restoration of effector T cells. Collectively, these findings positioned CD39 as a critical regulator of immune exhaustion and dysregulation in TB, with age-dependent effects, and a potential biomarker and therapeutic target for reversing immune dysfunction and enhancing antimicrobial immunity in fighting against TB.

Consistent with established TB research in whole blood or PBMCs (28, 29, 51), our findings demonstrated distinct upregulated expression patterns of multiple IRs, including LAG-3, CD39, CD160, and VISTA, in active TB patients compared to TBI and HC individuals. In contrast, CD39 expression showed no significant difference in TB lung tissues versus adjacent normal tissues, suggesting a systemic rather than local tissue-specific role for CD39 in TB pathogenesis. In addition, CD39 expression was elevated in older TB patients relative to younger cases. While prior studies have demonstrated CD39’s expression on Tregs and its potential role in suppressing Th1 responses in elderly TB patients (27, 52), our analysis did not find increased Treg infiltration in older TB patients, suggesting that the age-associated CD39 upregulation may not be solely driven by Tregs. Our age-stratified analysis indicated complex CD39 associations with neutrophils, memory T cells, monocytes, and macrophages across different age groups, pointing to multifaceted, age-dependent immunoregulatory functions. Our study further demonstrated that peripheral blood CD39 mRNA could serve as a potential diagnostic marker for active TB, exhibiting superior accuracy compared to IFN-γ, especially when combined with TB-antigen-responsive genes like TBX21 and GZMB. Due to critical limitations in current diagnostic tools, diagnoses are often delayed or inaccurate, particularly in resource-limited settings (53). For example, sputum smear microscopy has limited sensitivity, particularly in individuals with paucibacillary disease, HIV co-infection, or those unable to produce sputum samples (54). IFN-γ release assays (IGRAs) may exhibit reduced sensitivity in immunocompromised individuals and young children, and they cannot reliably distinguish between active TB disease and TBI (54). In contrast, CD39, potentially in combination with TBX21 and GZMB, might serve as a minimally invasive, blood-based diagnostic marker for active TB, offering a transformative alternative to traditional methods. Specifically, CD39 mRNA levels could be readily assessed from whole blood or PBMC samples using quantitative reverse transcription PCR (qRT-PCR), a relatively inexpensive and widely available technique (55). Alternatively, CD39 surface expression on circulating immune cells could be quantified using flow cytometry, allowing for rapid and multiparametric analysis of cell subsets expressing CD39 (27–29). Given the increasing accessibility and decreasing cost of RNA sequencing (RNA-seq), CD39 expression, alongside other relevant biomarkers, could also be incorporated into broader transcriptomic diagnostic panels (56, 57). The ability to leverage these various platforms makes CD39 a highly adaptable diagnostic candidate, although its elevation in select other infections (e.g., MRSA, *S. pneumoniae*, chronic HBV) warrants context-specific interpretation, emphasizing the necessity of considering the overall clinical picture and the potential for combinatorial biomarker approaches in diagnostic applications. However, comprehensive validation in prospective clinical cohorts, coupled with rigorous standardization of assay protocols, is essential to translate this potential into practical application.

Cytokine-mediated immunity, particularly Th1 responses characterized by the production of IFN-γ and IL-2, is critical for controlling Mtb infection (58). However, excessive inflammation and dysregulation of the cytokine network can contribute to disease progress and immunopathology. It has been reported that CD39 pathway could suppress Th1 cell function in TB, with CD39^+^ CD4^+^ T cells exhibiting exhausted phenotypes, impaired Th1 cytokine production, and heightened apoptosis (29). Consistent with this study (29), we observed a negative correlation between CD39 levels and the Th1 cytokine IFN-γ in TB patients. Paradoxically, we found CD39-high patients had higher levels of IL-2. A possible contributing factor to this observation may be the dynamic change of IL-2 levels in Mtb infection, where IL-2 levels have been shown to decrease with the extension of infection time (59). Our findings linked high CD39 to a Th2/Th17-polarized environment and neutrophil-mediated inflammation, suggesting that CD39-driven adenosine signaling might plays a critical role in skewing the immune response toward Th2/Th17 dominance while suppressing Th1 immunity. This shift could foster an immunosuppressive niche conducive to Mtb persistence. While elevated IL-17A and IL-22 in CD39-high patients might suggest initial compensatory mechanisms aimed at reinforcing mucosal barriers, given that excessive Th17 activity is known to correlate with neutrophil recruitment and subsequent tissue damage (60), this apparent compensatory response could paradoxically contribute to pathology. Indeed, even though neutrophils are critical for early bacterial containment, they can also drive unresolved inflammation and cavity formation through neutrophil extracellular trap (NET) formation and matrix metalloproteinase (MMP)-mediated tissue damage (61). Our observation that high CD39 expression exhibited increased levels of Th17 cytokines, pronounced neutrophil infiltration, and a positive correlation with neutrophil effector genes (ELANE and MPO) strongly supports a mechanistic link between CD39, Th17 responses, and neutrophil-mediated pathology in TB. In addition, we observed that memory B cells and plasma cells were reduced in CD39-high patients, likely due to adenosine-mediated suppression of B-cell receptor (BCR) signaling via cAMP/PKA-dependent inhibition of NF-κB activation in antigen-stimulated B cell models (62). This reduction might impair antibody-mediated immunity and antigen presentation, disrupting bacterial opsonization and macrophage-mediated clearance, thereby perpetuating Mtb persistence (63).

The spatiotemporal dynamics of CD39 during Mtb infection, as revealed by single-cell transcriptomic profiling of non-human primate granulomas, highlight a complex and potentially contradictory role in shaping the immune response. We found that while CD39 expression initially localized to specific immune cell subsets, it became more widespread across diverse immune cell populations between 4 and 10 weeks post-infection. However, this expanded cellular distribution coincided with a decline in CD39 transcriptional activity within individual cell types. This seemingly contradictory pattern suggested that the role of CD39 might evolve as the immune response transitions from an initial, localized phase to a more chronic and systemic state. Early infection (4-week) showed CD39 expression primarily in macrophages and T cells, potentially reflecting its role in modulating macrophage activation states and driving rapid T cell terminal differentiation (64, 65), which was in line with our trajectory analysis demonstrating that CD39^+^ T cells predominantly reach terminal states at this stage. By contrast, in prolonged infections at 10 weeks, this pro-differentiation function appeared to be suppressed, characterized by declining transcriptional activity of CD39. Instead, the broader expression of CD39 across diverse cell types might reflect its role in promoting immune exhaustion and widespread immune suppression in chronic infections (66, 67). To resolve this functional dichotomy, future studies should leverage spatial transcriptomics of human granulomas to map CD39-dependent cellular crosstalk, specifically investigating whether its shift from pro-differentiation to immunosuppressive roles during chronic infection orchestrates distinct ligand-receptor networks within the TB microenvironment.

Therapeutically, we found that anti-TB drug treatment induced downregulation of CD39, ultimately restoring CD39 levels to those observed in HC individuals after successful treatment. In line with these results, other studies demonstrated a gradually declined level of CD39 and other IRs (e.g., BTLA and PD-L1) during anti-TB treatment (29, 68). This reduction was accompanied by shifts in immune cell populations, favoring cells associated with effective immunity and decreased numbers of immunosuppressive cells, suggesting a broader restoration of immune competence. Early in treatment, inflammatory modulation pathways are enriched, followed by enrichment of tissue repair pathways after long-term therapy. These findings suggested that CD39 reduction post-treatment may reflect a systemic restoration of immune competence, with CD39 potentially serving as a biomarker for treatment response and immune reconstitution in TB patients. Targeting CD39 may reverse immunosuppression and enhance pathogen clearance. However, risks require cautious consideration: CD39 inhibition might exacerbate neutrophilic inflammation (e.g., NETosis), and adenosine blockade could impair its protective role in curbing excessive immune activation. Combining CD39 inhibitors with Th2/Th17 pathway modulation may synergistically restore immune equilibrium, representing a critical focus for future research.

As a bioinformatics study primarily relying on publicly available data, our conclusions are subject to certain limitations. While we identified strong correlations between CD39 and neutrophil infiltration, *in vitro*/*in vivo* mechanistic studies are needed to clarify whether CD39 directly drives neutrophil recruitment or reflects compensatory inflammation. Similarly, the causal relationship between CD39 and Th2/Th17 skewing warrants investigation using genetic knockout/inhibition models. Our reliance on bulk RNA-seq data, while providing valuable insights, might obscure cell-specific CD39 roles and dynamic interactions within complex immune environments, necessitating single-cell resolution validation and functional assays. Furthermore, ethnic and age-related biases in public datasets limited the generalizability of our findings. These limitations highlight critical needs for future research: 1) Cross-cohort validation in diverse populations to verify CD39’s diagnostic robustness across demographics and geographic settings; 2) Multi-omics integration (e.g., proteomics/metabolomics) to substantiate transcriptional findings and uncover CD39’s mechanistic roles; 3) Prospective clinical trials validating CD39’s diagnostic utility, particularly in combination with TB-antigen-responsive genes; and 4) Preclinical assessment of CD39 inhibitors in Mtb-infected models to assess therapeutic potential.

## Conclusion

In conclusion, our integrated analysis of publicly available datasets positioned CD39 as a pivotal regulator of TB-associated immune exhaustion and neutrophil-driven inflammation, with demonstrated superior diagnostic accuracy and strong therapeutic potential signaled by its downregulation during successful treatment. CD39’s roles were spatiotemporally dynamic, showing age-dependent immunoregulatory functions, distinct expression patterns across infectious diseases, and cell-specific redistribution with declining transcriptional activity during chronic granuloma formation. While our bioinformatic approach identified robust correlations across disease states, treatment response, and immune landscapes, mechanism validation in preclinical models is critical to elucidating the cell-specific role of CD39 in driving immune dysregulation and promoting Mtb persistence. Future studies should prioritize delineating the spatiotemporal regulation of CD39 in TB granulomas, exploring its interactions with various immune cell subsets, and validating CD39-targeted therapies through preclinical models and, ultimately, randomized clinical trials to advance translational applications for TB diagnosis and treatment. Furthermore, investigating the precise mechanisms underlying age-related differences in CD39 expression and function will be crucial for developing personalized therapeutic strategies targeting this key immunoregulatory molecule.

## Data Availability

The datasets presented in this study can be found in online repositories. The names of the repository/repositories and accession number(s) can be found in the article/**Supplementary Material**. For any further inquiries, please contact the corresponding author.

## Glossary

AFB: acid-fast bacilli
APCs: antigen-presenting cells
AUC: Area Under the Curve
BCG: Bacillus Calmette-Guérin
BCR: B-cell receptor
CI: Confidence intervals
COVID-19: coronavirus disease 2019
CTLA-4: Cytotoxic T-lymphocyte-associated protein 4
DCs: dendritic cells
DS: drug-sensitive
ENTPD1: ectonucleoside triphosphate diphosphohydrolase-1
EPTB: extrapulmonary TB
GSEA: Gene Set Enrichment Analysis
HBV: hepatitis B virus
HC: healthy controls
IRs: inhibitory receptors
ITIM: immunoreceptor tyrosine-based inhibitory motif
LAG-3: lymphocyte activation gene 3
LRTI: lower respiratory tract infection
TBI: TB infection
MDR: multidrug-resistant
Mtb: *Mycobacterium tuberculosis*
MMP: matrix metalloproteinase
MRSA: methicillin-resistant *Staphylococcus aureus*
MSSA: methicillin-sensitive *S. aureus*
NET: neutrophil extracellular trap
PD-1: programmed cell death protein 1
PTB: pulmonary TB
SCC: sputum culture conversion
scRNA-seq: single-cell RNA sequencing
TB: tuberculosis
TCR: T-cell receptor
TIGIT: T cell immunoreceptor with immunoglobulin and immunoreceptor tyrosine-based inhibitory domains
TIM-3: T cell immunoglobulin and mucin domain-containing-3
Tregs: regulatory T cells
ROC: Receiver Operating Characteristic
UMAP: uniform manifold approximation and projection
URTI: upper respiratory tract infection
VISTA: V-domain Ig suppressor of T-cell activation
XDR: extensively drug-resistant
