# Supplementary material for: CD39 dynamics in tuberculosis: a potential biomarker of immune dysregulation and T cell exhaustion
Source: Front Immunol. 2025 Aug 11;16:1601637. doi: 10.3389/fimmu.2025.1601637 (PMC12375674; doi:10.3389/fimmu.2025.1601637)
Supplement: Supplementary file 1 [file DataSheet1.pdf]

Supplementary Materials

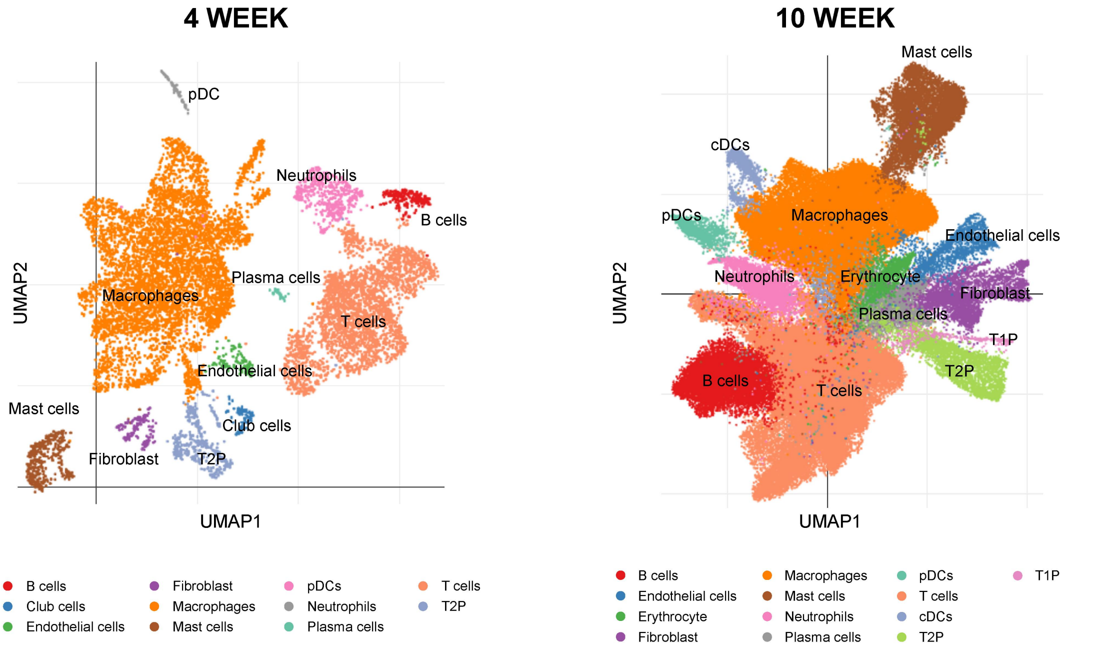

Figure S1. UMAP projection for cell identity of scRNA-seq data (SCP257 and SCP1749 studies) at two infection time points.

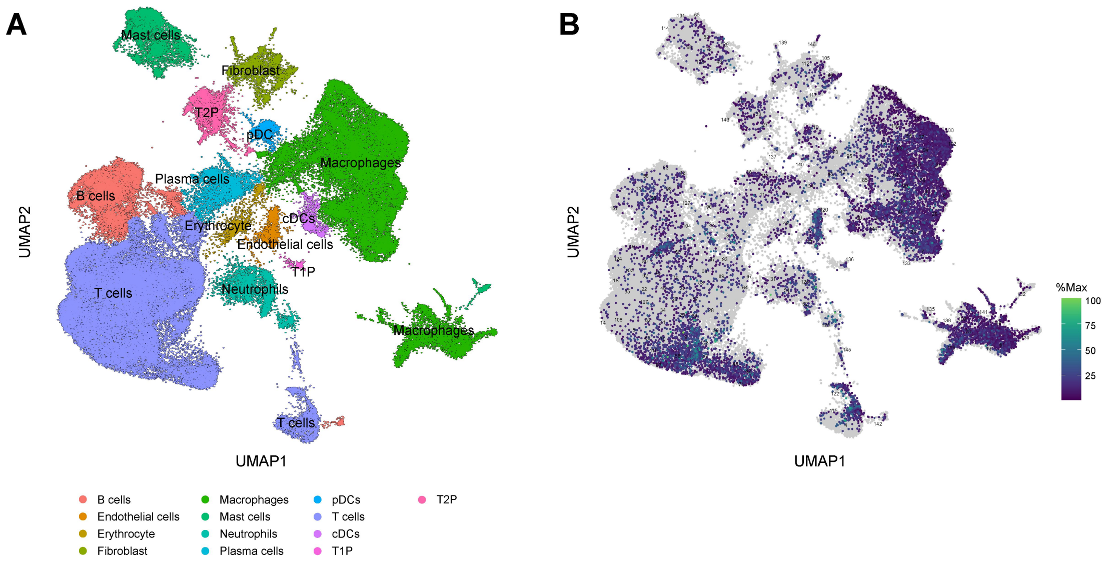

Figure S2. Integrated UMAP projection of SCP257 and SCP1749 scRNA-seq data, depicting cell identities (A) and CD39 expression patterns (B) across the combined infection time points.

Table S1. Pathways significantly enriched in gene expression profiles associated with elevated CD39 levels in TB patients from the GSE83456 dataset.

Details	Size	ES	NES	NOM p-val	FDR q-val
REACTOME_NEUTROPHIL_DEGRANULATION	462	0.582	3.505	0	0
WP_TYROBP_CAUSAL_NETWORK_IN_MICROGLIA	58	0.655	3.018	0	0
WP_IL4_SIGNALING	54	0.647	2.981	0	0
REACTOME_SIGNALING_BY_CSF3_G_CSF	30	0.748	2.979	0	0
WP_IMMUNE_RESPONSE_TO_TUBERCULOSIS	22	0.798	2.947	0	0
WP_MICROGLIA_PATHOGEN_PHAGOCYTOSIS_PATHWAY	39	0.690	2.926	0	0
WP_TOLLLIKE_RECEPTOR_SIGNALING_RELATED_TO_MYD88	30	0.720	2.913	0	0
WP_LEPTIN_SIGNALING	76	0.589	2.897	0	0
WP_IL6_SIGNALING	43	0.651	2.891	0	0
REACTOME_ANTIGEN_PROCESSING_CROSS_PRESENTATION	96	0.559	2.838	0	0

Table S2. Pathways significantly enriched in gene expression profiles at 4 weeks post-TB treatment compared to baseline in the GSE31348 dataset.

Details	Size	ES	NES	NOM p-val	FDR q-val
PID_IL8_CXCR2_PATHWAY	29	0.577	2.054	0	0.197
PID_KIT_PATHWAY	49	0.494	1.985	0	0.225
PID_RAC1_PATHWAY	50	0.488	1.968	0	0.183
KEGG_MEDICUS_REFERENCE_TYPE_I_IFN_SIGNALING_PATHWAY	22	0.587	1.946	0.002	0.180
REACTOME_OLFACTORY_SIGNALING_PATHWAY	94	0.413	1.936	0	0.160
KEGG_MEDICUS_REFERENCE_TYPE_I_INTERFERON_TO_JAK_STAT_SIGNALING_PATHWAY	22	0.586	1.929	0.002	0.146
KEGG_MEDICUS_REFERENCE_TYPE_II_INTERFERON_TO_JAK_STAT_SIGNALING_PATHWAY	21	0.576	1.883	0.004	0.208
WP_HOSTPATHOGEN_INTERACTION_OF_HUMAN_CORONAVIRUSES_AUTOPHAGY	17	0.621	1.869	0.002	0.210
KEGG_MEDICUS_REFERENCE_TLR7_8_9_IRF5_SIGNALING_PATHWAY	22	0.549	1.842	0.004	0.246
SIG_BCR_SIGNALING_PATHWAY	45	0.461	1.834	0.002	0.237

Table S3. Pathways significantly enriched in gene expression profiles at 26 weeks post-TB treatment compared to baseline in the GSE31348 dataset.

Details	Size	ES	NES	NOM p-val	FDR q-val
PID_DNA_PK_PATHWAY	15	0.648	1.945	0	0.630
KEGG_FRUCTOSE_AND_MANNOSE_METABOLISM	32	0.518	1.907	0	0.470
WP_INTRACELLULAR_TRAFFICKING_PROTEINS_INVOLVED_IN_CMT_NEUROPATHY	25	0.513	1.827	0	0.684
REACTOME_TBC_RABGAPS	41	0.456	1.741	0	1
BIOCARTA_NKT_PATHWAY	24	0.507	1.740	0.003	0.861
WP_INFLAMMATORY_RESPONSE_PATHWAY	30	0.475	1.728	0.006	0.792
REACTOME_INTERLEUKIN_RECEPTOR_SHC_SIGNALING	27	0.497	1.720	0.008	0.723
WP_BMP_SIGNALING_IN_EYELID_DEVELOPMENT	20	0.504	1.674	0.024	0.904
REACTOME_INTERLEUKIN_3_INTERLEUKIN_5_AND_GM_CSF_SIGNALING	48	0.411	1.654	0.003	0.932
REACTOME_REGULATION_OF_ENDOGENOUS_RETROELEMENTS_BY_KRAB_ZFP_PROTEINS	44	0.411	1.652	0.009	0.855

Table S4. Pathways significantly enriched in gene expression profiles at 3 months post-TB treatment compared to baseline in the GSE54992 dataset.

Details	Size	ES	NES	NOM p-val	FDR q-val
WP_TAMOXIFEN_METABOLISM	18	0.769	1.944	0	0.093
WP_CYTOKINES_AND_INFLAMMATORY_RESPONSE	26	0.654	1.773	0	0.743
WP_THYROXINE_THYROID_HORMONE_PRODUCTION	24	0.645	1.755	0	0.642
BIOCARTA_INFLAM_PATHWAY	26	0.648	1.744	0.002	0.561
KEGG_MEDICUS_REFERENCE_CCR_CXCR_GNB_G_PI3K_RAC_SIGNALING_PATHWAY	26	0.619	1.688	0.005	0.906
REACTOME_INWARDLY_RECTIFYING_K_CHANNELS	34	0.576	1.681	0.007	0.826
KEGG_MEDICUS_REFERENCE_CA2_ENTRY_VOLTAGE_GATED_CA2_CHANNEL	25	0.620	1.660	0.011	0.903
KEGG_MEDICUS_REFERENCE_CARDIAC_TYPE_VGCC_RYR_SIGNALING	21	0.637	1.649	0.018	0.890
REACTOME_CELLULAR_HEXOSE_TRANSPORT	17	0.671	1.644	0.011	0.829
REACTOME_KERATINIZATION	118	0.458	1.619	0.003	0.988

Table S5. Pathways significantly enriched in gene expression profiles at 6 months post-TB treatment compared to baseline in the GSE54992 dataset.

Details	Size	ES	NES	NOM p-val	FDR q-val
KEGG_MEDICUS_REFERENCE_TRANSLATION_INITIATION	74	0.558	2.420	0	0
WP_CYTOKINES_AND_INFLAMMATORY_RESPONSE	26	0.631	2.151	0	0.030
WP_MELATONIN_METABOLISM_AND_EFFECTS	33	0.574	2.080	0	0.056
BIOCARTA_CYTOKINE_PATHWAY	18	0.673	2.050	0	0.063
REACTOME_INTERLEUKIN_10_SIGNALING	41	0.535	1.997	0	0.091
BIOCARTA_INFLAM_PATHWAY	26	0.584	1.967	0	0.108
WP_PHOTODYNAMIC_THERAPYINDUCED_NFKB_SURVIVAL_SIGNALING	33	0.555	1.966	0	0.093
WP_IMMUNE_INFILTRATION_IN_PANCREATIC_CANCER	36	0.517	1.884	0.003	0.195
BIOCARTA_IL1R_PATHWAY	29	0.522	1.858	0.004	0.223
BIOCARTA_CTLA4_PATHWAY	20	0.603	1.852	0	0.211

Table S6. Pathways significantly enriched in gene expression profiles at 12 months post-TB treatment compared to baseline in the GSE19435 dataset.

Details	Size	ES	NES	NOM p-val	FDR q-val
REACTOME RRNA MODIFICATION IN THE NUCLEUS AND CYTOSOL	57	0.651	2.163	0	0
WP MRNA PROCESSING	123	0.576	2.128	0	0.001
REACTOME PROCESSING OF CAPPED INTRON CONTAINING PRE MRNA	271	0.543	2.125	0	0.001
REACTOME RRNA PROCESSING	198	0.550	2.094	0	0.001
REACTOME TRNA PROCESSING	103	0.581	2.088	0	0.001
KEGG MEDICUS REFERENCE NUCLEAR EXPORT OF MRNA	30	0.686	2.049	0	0.001
KEGG DNA REPLICATION	36	0.674	2.049	0	0.001
REACTOME TRANSPORT OF MATURE TRANSCRIPT TO CYTOPLASM	78	0.584	2.043	0	0.001
REACTOME TRNA MODIFICATION IN THE NUCLEUS AND CYTOSOL	42	0.646	2.035	0	0.001
KEGG SPLICEOSOME	126	0.538	1.968	0	0.004
